# Impact of climate change on spontaneous abortion: a systematic review and meta-analysis

**DOI:** 10.3389/fgwh.2026.1709985

**Published:** 2026-03-20

**Authors:** Dessie Abebaw Angaw, Betelhem Abebe Andargie, Nimrod Muhumuza, Gertrude Nakanwagi, Moses Mulumba

**Affiliations:** 1 Evidence Generation Department, Afya na Haki Institute, Kampala, Uganda; 2 Department of Epidemiology and Biostatistics, Institute of Public Health, College of Medicine and Health Sciences, University of Gondar, Gondar, Ethiopia; 3 Afya na Haki Institute, Kampala, Uganda

**Keywords:** abortion, climate change, meta-analysis, miscarriage, systematic review

## Abstract

**Purpose:**

This systematic review and meta-analysis was conducted to synthesize the existing evidence on the association between climate change-related factors and spontaneous abortion.

**Patients and method:**

We conducted a systematic literature search across PubMed, Embase, MEDLINE, Web of Science, PsycINFO, and Google Scholar for studies published in English. Eligible studies included all observational study designs that assessed the relationship between climate-related exposures and spontaneous abortion. Quality assessment was performed using the Newcastle–Ottawa Scale. Random-effects models were applied to pool effect estimates, expressed as odds ratios (ORs) with 95% confidence intervals (CIs). Subgroup and sensitivity analyses were conducted to explore heterogeneity.

**Results:**

Of the 19,167 records, 37 studies met the eligibility criteria. Pooled estimates demonstrated significant positive associations between maternal exposure to climate-related factors, including air pollution, high ambient temperature, floods, and wildfire smoke, and spontaneous abortion: increased risks were observed with exposure to sulfur dioxide (OR = 1.60, 95% CI: 1.20–2.13), ozone (OR = 1.27, 95% CI: 1.01–1.60), carbon monoxide (OR = 1.48, 95% CI: 1.06–2.06), nitrogen dioxide (OR = 1.17 95% CI: 1.00–1.38), particulate matter (OR = 1.15, 95% CI: 1.06–1.24), and high ambient temperature/heat exposure (OR = 1.39, 95% CI: 1.07–1.79). Subgroup analyses showed that exposure to high temperature was associated with an elevated risk of spontaneous abortion among women living in lower-middle-income countries and abortions before 20 weeks of gestation. Evidence on floods and wildfire smoke exposure also suggested an increased risk of spontaneous abortion.

**Conclusion:**

Climate change-related exposures are significantly associated with an increased risk of abortion, particularly spontaneous abortion. These findings underscore the urgent need to integrate reproductive health into climate adaptation strategies, strengthen surveillance systems, and develop climate-resilient health infrastructures to protect vulnerable populations.

**Systematic Review Registration:**

https://www.crd.york.ac.uk/PROSPERO/view/CRD420251120772, PROSPERO CRD420251120772.

## Introduction

Climate change refers to long-term alterations in temperature and weather patterns. Although such changes have occurred naturally, the current trend is primarily the result of human activities (1). Climate change has emerged as one of the greatest public health challenges of the 21st century, with wide-ranging impacts on human health and survival (2). Air pollution, long recognized as an environmental hazard, is increasingly shaped by climate change through shared emission sources and climate-driven processes that intensify the formation and accumulation of harmful pollutants such as particulate matter and ozone (3).

Globally, an estimated 3.6 billion people live in regions that are highly vulnerable to the impacts of climate change (4, 5). Despite contributing minimally to global greenhouse gas emissions, developing countries bear the highest share of the resulting health burdens. The impact on maternal health is particularly significant. Climate change exacerbates existing gender inequalities and directly affects women's sexual and reproductive health by increasing health risks and limiting access to healthcare services (6, 7).

Climate change can also lead to increased rates of abortion, both directly through exposure to extreme heat and indirectly, by contributing to unintended pregnancies, gender-based violence, and displacement caused by climate-related events (8). Globally, approximately 73 million induced abortions occur each year, of which 45% are considered unsafe (9, 10). Abortion refers to the expulsion of a fetus from the uterus before it becomes viable. It may occur spontaneously due to factors such as maternal illness, trauma, genetic defects, or maternal–fetal incompatibility. Alternatively, it may be intentionally induced (11). Evidence suggests that exposure to high temperatures caused by climate change during early pregnancy is associated with an increased risk of spontaneous abortion and pregnancy loss (12–14).

Marie Stopes International (MSI) Reproductive Choices reports that climate-related displacement has disrupted contraceptive access for 11.5 million women in climate-affected countries, with an additional 14 million women projected to lose access over the next decade, potentially leading to 2.1 million unsafe abortions (15). Furthermore, in low- and middle-income countries, where healthcare systems are already weakened and disrupted, climate change further overburdens these systems and reduces access to health services, contributing to the rise in unsafe abortion (16, 17).

To reduce the impact of climate change on maternal, sexual, and reproductive health, international organizations have launched various interventions and strategies. For instance, governments world have been requested to advance their National Adaptation Plans (NAPs) to address the impacts of climate change on sexual and reproductive health issues (18). In 2023, the World Health Organization (WHO), the United Nations Children's Fund (UNICEF), and the United Nations Population Fund (UNFPA) issued a joint call to action, emphasizing the need for urgent action and incorporating the needs of pregnant women, babies, and children into climate and disaster-related policies. These agencies also call for further research to better understand the impacts of climate change on maternal and child health (19).

Given the accelerating pace of climate change and the projected amplification of extreme weather events, the potential for widening reproductive health inequities is substantial. To date, evidence examining the relationship between climate change-related factors and abortion remains limited. Understanding the magnitude and direction of climate change-related effects on abortion can provide policymakers with actionable evidence to design climate-resilient reproductive health interventions. In addition, synthesizing findings across exposures and geographies can help identify which climate-related stressors pose the greatest risks. Understanding these dynamics can guide the integration of reproductive health into climate adaptation and mitigation strategies, ensuring that the needs of women are not overlooked in climate policy agendas.

Therefore, this systematic review and meta-analysis aimed to synthesize evidence on the impact of climate change on abortion. Specifically, this review sought to address the following key questions:

1. What is the association between climate change-related exposures and spontaneous abortion?
2. Is there any heterogeneity by income group, gestational age, or exposure measurement methods?

## Methods

### Study design and registration

This systematic review and meta-analysis was conducted in accordance with the Preferred Reporting Items for Systematic Reviews and Meta-Analysis (PRISMA) 2020 guidelines (20). The study protocol was registered in the International Prospective Register of Systematic Reviews (PROSPERO, CRD420251120772), ensuring methodological transparency and rigor.

### Eligibility criteria

We included all published observational studies that assessed the relationship between climate change-related factors and abortion. The population of interest included women of reproductive age. Regarding exposure, studies examining both direct and indirect effects of climate change were eligible. These included extreme weather events (such as floods, heat waves, and storms), shifts in temperature or precipitation patterns, and air pollution. Studies with or without comparator groups were included. For the outcome, any study reporting on abortion, including both induced and spontaneous abortion (miscarriage), was considered. Studies from all publication years and geographic regions were eligible for inclusion. However, only studies published in English were included.

### Information source and search strategy

A comprehensive literature search was conducted across electronic databases, including PubMed, Embase, Web of Science, MEDLINE, PsycINFO, and Google Scholar. In addition, the reference lists of included articles were reviewed, and citation tracking (snowballing) was performed to identify additional relevant studies. The search strategy included the keywords identified in the study and a mixture of MeSH terms. Entry terms were identified using the MeSh browser and through brainstorming (Supplementary File 1). Search terms included combinations of keywords related to climate change and abortion. Boolean operators (AND, OR) and truncation symbols were used to combine search terms and capture variations in terminology. All databases were searched up to 24 July 2025.

### Study selection

All citations identified during the literature search and reference list screening were exported to EndNote 7 citation management software, and duplicate records were removed. Two reviewers independently screened the titles and abstracts. The full texts of potentially relevant studies were retrieved and assessed independently by the same reviewers. Any discrepancies regarding study inclusion or exclusion were resolved through discussion, and, if necessary, a third reviewer was consulted to reach consensus. The selection process is illustrated using a PRISMA flowchart.

### Data extraction

Data were extracted from all included studies using a prepiloted data extraction form. Two reviewers independently extracted the data from each included study. Any discrepancies in data extraction were resolved through discussion between the two reviewers. Data items on study characteristics (author, year, setting, continent, country income level, study objective, study design, outcome sample size, study population), exposure characteristics (type of climate change-related exposures, exposure assessment methods), confounder adjusted for in the analysis, effect estimates with 95% confidence intervals (CIs), outcome characteristics (abortion type, gestational age, outcome measurement), and study quality were extracted. The extracted data were summarized in tabular form and visually displayed to summarize individual study characteristics and findings.

### Outcome measurement

The primary outcome of this study was spontaneous abortion/miscarriage, defined as non-induced pregnancy loss before fetal viability.

### Quality appraisal

The methodological quality of the included studies was assessed independently by two reviewers. Any disagreements between the reviewers were resolved through discussion or by consulting a third reviewer if no consensus could be reached. As all included studies were observational in design, the Newcastle–Ottawa Scale (NOS), which assigns a star-based rating ranging from 0 to 9, was used to assess their quality. Studies scoring 7–9 were categorized as high quality, those scoring 4–6 as medium quality, and those scoring 0–3 as low quality (21).

### Data synthesis and analyses

Given the heterogeneity in climate-related exposure types, study designs, and geographic settings, this review primarily utilized a comprehensive narrative synthesis to integrate and interpret the findings. Studies were organized thematically according to the type of climate-related exposure. Where sufficient quantitative data were available, we conducted meta-analyses using random-effects models to account for between-study variability. Effect sizes were expressed as odds ratios (ORs) with 95% CIs. For consistency, the relative risks reported in four studies were converted to ORs under the rare disease assumption (22). Statistical heterogeneity was assessed using the Q statistic (with *p* < 0.05 indicating heterogeneity) and quantified using the *I*^2^ statistic; I² values were interpreted as follows: 0%–25% (low heterogeneity), 25%–50% (moderate heterogeneity), 50%–75% (substantial heterogeneity), and >75% (significant heterogeneity) (23). For studies that showed substantial heterogeneity, subgroup analyses were performed according to country income level, pregnancy window, and exposure measurement methods to explain the variability between studies. To ensure robustness, sensitivity analyses were also performed using a leave-one-out method to determine the influence of individual studies. Finally, when sufficient studies (≥10) reporting the same exposures were available, publication bias was assessed using the funnel plot and Egger's test for small-study effects. All quantitative analyses were performed using Stata software.

### Ethical considerations

As this review was conducted using publicly available data from published literature, no ethical approval was required.

## Results

### Study selection

**Figure 1:**
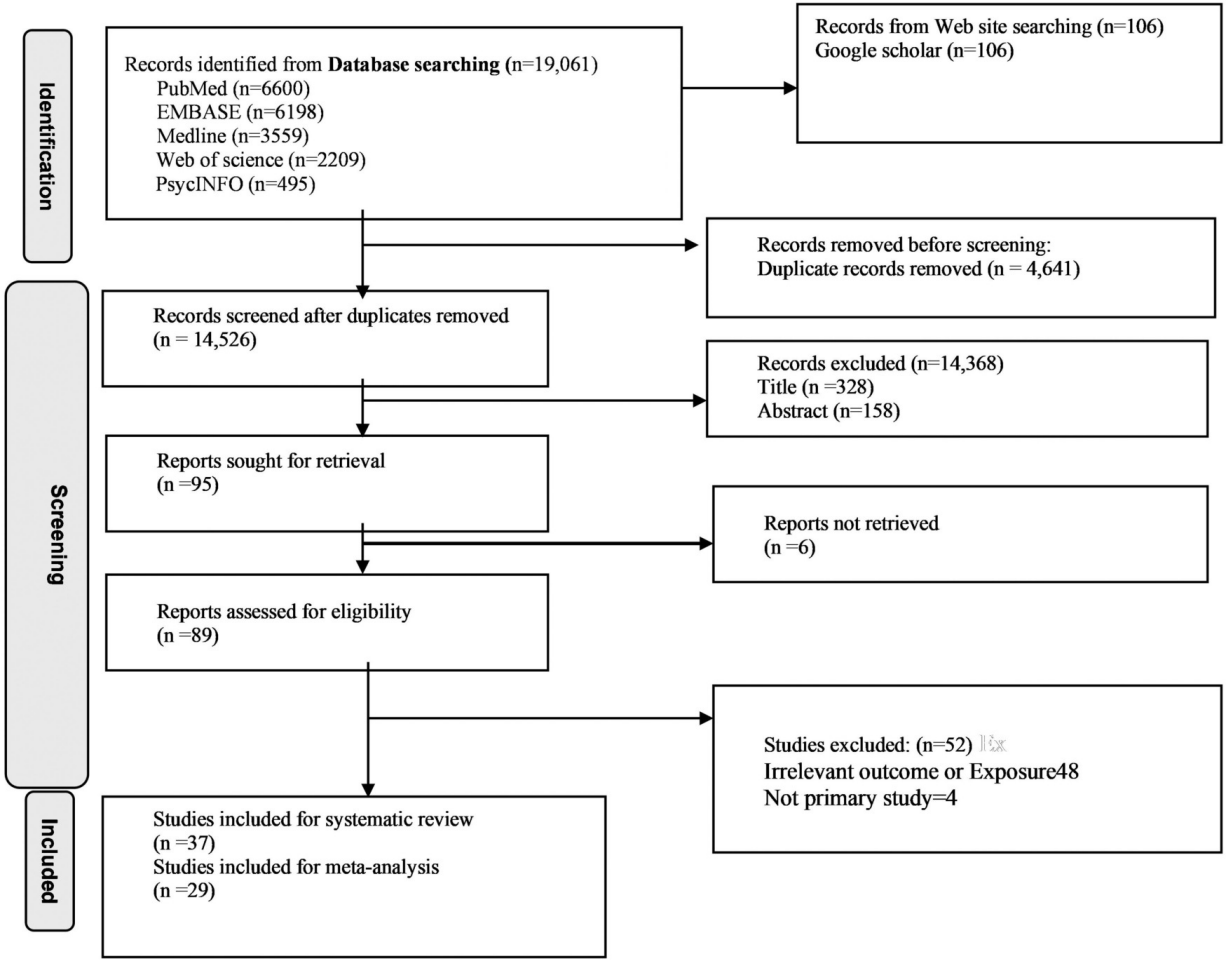
Flow diagram of search results, study screening, and study inclusion.

A total of 19,167 articles were identified in the literature search. After removing 4,641 duplicate records, 14,526 articles remained for screening. After screening titles and abstracts, 14,368 articles were excluded, including commentaries, reviews, case reports, and animal studies. After full-text assessment of 89 studies, 52 were excluded due to irrelevant outcomes or exposures (*n* = 48) and because they were not primary studies (*n* = 4) (Supplementary File 2). Finally, 37 studies met the inclusion criteria for the systematic review, of which 29 were included in the meta-analysis (Figure 1).

### Study characteristics

**Figure 2:**
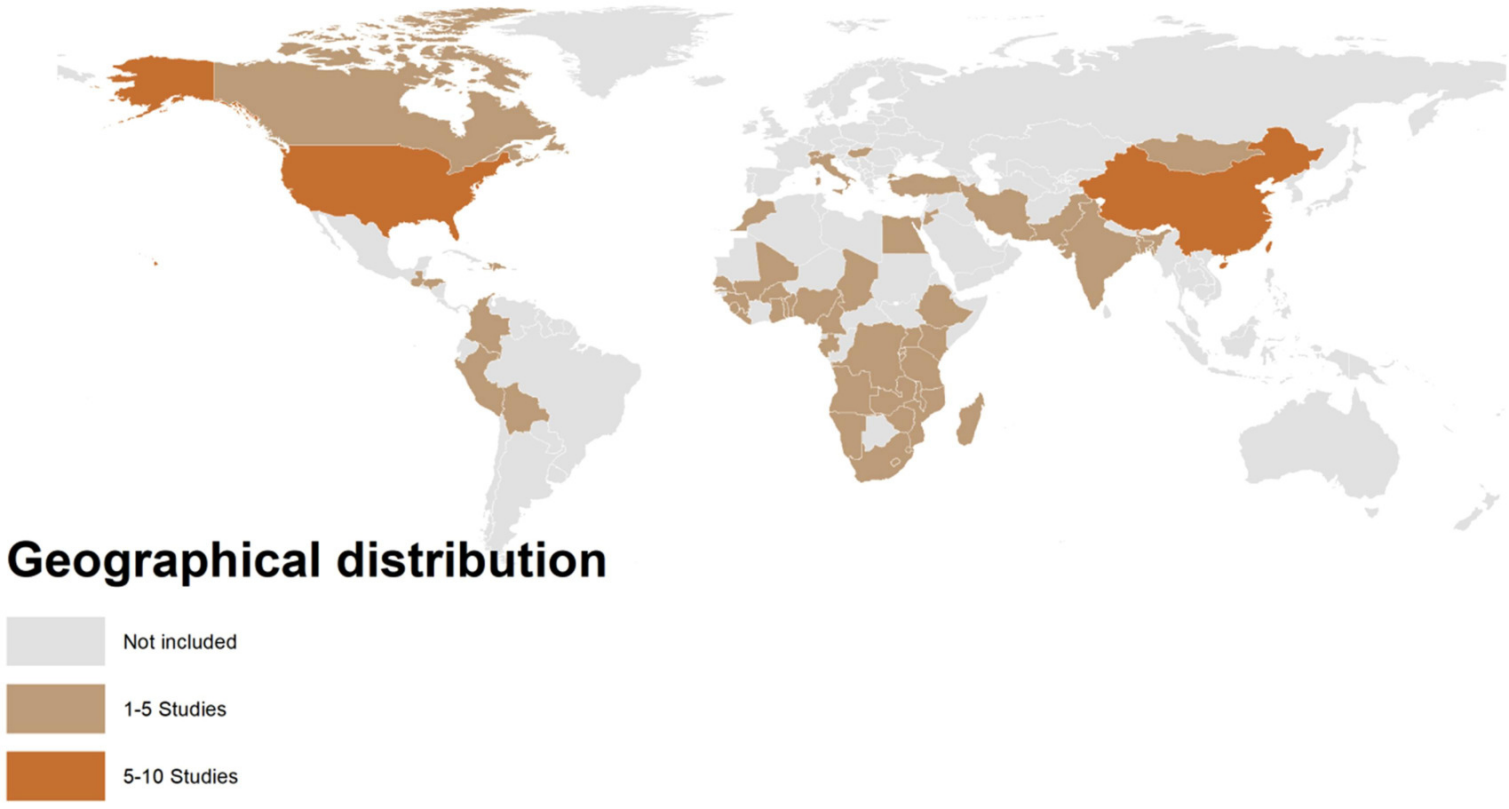
Geographical distribution of included studies.

A total of 37 studies published between 2001 and 2025 were included in this review (Table 1). These studies were conducted across four continents: Asia, North America, Europe, and Africa. In Asia, the majority of studies were conducted in China (*n* = 10) (12, 25, 34–36, 38, 42, 43, 46, 50, 53), followed by Iran (*n* = 4) (26, 27, 44, 47), Bangladesh (*n* = 1) (48), India (*n* = 1) (51), Mongolia (*n* = 1) (28), and one in South Asia (India, Pakistan, and Bangladesh) (*n* = 1) (40). In North America, seven studies were conducted in the United States (*n* = 7) (13, 31, 32, 37, 41, 45, 55), and two studies included populations from both the United States and Canada (24). In Europe, one study each was conducted in Switzerland (*n* = 1) (56), Italy (*n* = 1) (29), Hungary (*n* = 1) (49), and Turkey (*n* = 1) (39). In Africa, studies were conducted in Ghana (*n* = 1) (30), South Africa (*n* = 1) (54), alongside a large multicountry analysis covering 33 African countries (*n* = 1) (57) and another analysis including 15 sub-Saharan African (SSA) countries (*n* = 1) (33). In addition, one multicountry study spanning 33 developing countries was included (52) (Figure 2). Regarding study design, case–control studies were the most frequent (*n* = 13) (12, 25–27, 34, 36, 38, 40, 49, 50, 52, 53, 57), followed by cohort studies (*n* = 12) (29, 31, 39, 44, 45, 56) and case-crossover, cross-sectional, or time-series studies (*n* = 10) (Table 1).

**Table 1:** Characteristics of included studies (*n* = 37).

Author, year	Study objective	Study setting	Study period	Study design	Participants	Main findings
Bianchi-Demicheli et al. (2001) (24)	To examine the influence of weather conditions on the incidence of outpatient emergency consultations at the Department of Obstetrics and Gynecology at the Regional Hospital	Switzerland	1 March 1995 to 29 February 1996	Cohort	385 women who attended the emergency outpatient	Meteorological factors, such as temperature and barometric pressure, appear to influence the probability of emergency admissions for gynecological symptoms, including threatened abortion
Hou et al. (2013) (25)	To examine the association between four air pollutants, namely, PM10, TSP, SO_2_, and NO_2_, and fetal loss in the first trimester in Tianjin, China	China	1 April 2001 to December 2006	Case–control	959 cases (spontaneous fetal loss) and 959 controls (normal intrauterine pregnancies)	The risk of first-trimester fetal loss was significantly affected by ambient SO_2_ and TSP air pollutant levels but not PM10 and NO_2_ levels
Thimonier et al. (2014) (26)	To evaluate the correlation between ambient concentrations of air pollutants and first-trimester spontaneous abortion	Iran	June 2010 to February 2011	Case–control	296 women, with 148 cases and 148 controls	Exposure to ambient air pollution during pregnancy, especially in the first trimester, may increase the risk of spontaneous abortion
Moridi et al. (2014) (27)	To evaluate the correlation between ambient concentrations of air pollutants and first-trimester spontaneous abortion	Iran	June 2010 to February 2011	Case–control study	296 women, with 148 cases and 148 controls	Ambient air pollution during pregnancy, especially in the first trimester, may increase the risk of spontaneous abortion
Enkhmaa et al. (2014) (28)	To examine the association between spontaneous abortion and the seasonal variation in air pollutants	Mongolia	2009–2011	Cross-sectional	Medical records of 1,219 women	There is a strong correlation between ambient levels of air pollutants and spontaneous abortion
Di Ciaula et al. (2015) (29)	To examine whether maternal exposure to specific air pollutants is associated with the occurrence of spontaneous abortions	Italy	1 January 2013 to 31 December 2013	Retrospective cohort	984 cases of spontaneous abortions	Spontaneous abortion occurrence is affected by PM10 and ozone concentrations
Asamoah et al. (2017) (30)	To test the hypothesis that maternal heat exposure during pregnancy in hot regions is associated with increased prevalence of spontaneous abortions or stillbirths	Ghana	2004–2007	Cross-sectional	1,136 women with pregnancy experiences	Environmental heat exposures may be associated with adverse pregnancy outcomes
Ha et al. (2018) (31)	To estimate the association of pregnancy loss with common air pollutant exposure	USA	2005–2009	Prospective cohort	343 singleton pregnancies	Chronic exposures to PM2.5 and ozone throughout the entire pregnancy are associated with pregnancy loss
Gaskins et al. (2019) (32)	To examine whether chronic exposure to particulate matter is associated with the risk of spontaneous abortion in a large, geographically diverse, prospective cohort of women	USA	1990–2008	Prospective cohort and case-crossover	35,025 pregnancies	There was a positive association between exposure to all size fractions of PM and risk of spontaneous abortion
Xue et al. (2019) (33)	To analyze the association between PM2.5 ​exposure and pregnancy loss, including miscarriage and stillbirth, in low-income countries in Africa	33 countries in Africa. The study region included Eastern, Middle-southern, Northern, and Western Africa	1 January 1998 to 31 December 2016	Case–control	42,952 pregnancy losses (miscarriage 30,418; stillbirth 12,534) matched to 107,910 successful deliveries	Gestational exposure to ambient PM2.5 was robustly related to an increased risk of pregnancy loss, particularly miscarriage, in Africa
Sun et al. (2019) (34)	To examine the association between maternal exposure to temperature and the risk of miscarriage and further assess the modifying effects of surrounding residential greenness	China	1 January 2014 and 31 December 2016	Hospital based case–control	2,044 cases of miscarriage and 2,285 controls	Maternal exposure to moderately high temperatures during pregnancy may increase the risk of miscarriage, but the modifying effects of greenness on these associations remain inconclusive
Zhang et al. (2019) (35)	To quantify the association between air pollution exposure and missed abortion in the first trimester in Beijing, China	China	2009–2017	Time series	255,668 pregnant women	An increased risk of missed abortion in the first trimester is associated with rises in pollutant concentrations for all four pollutants studied. The risk increase is not linear but becomes more severe at higher pollutant concentrations
Zhang et al. (2019) (36)	To estimate associations between clinically recognized early pregnancy loss and exposure to ambient PM2.5 during the peri-conception period	China	July 2017 to July 2018	Case–control	364 cases and 364 controls, for a total of 728 participants	Clinically recognized early pregnancy loss was associated with maternal acute exposures to ambient PM2.5
Leiser et al. (2019) (37)	To investigate the relationship between acute exposure to air pollutants and spontaneous pregnancy loss	USA	2007–2015	Case-crossover	1,398 spontaneous pregnancy loss events	Short-term exposure to elevated levels of air pollutants was associated with a higher risk for spontaneous pregnancy loss; particularly, NO_2_, is associated with spontaneous pregnancy loss
Davenport et al. (2020) (33)	To examine the link between climate (temperature and precipitation) and pregnancy outcomes, including miscarriages, stillbirths, and low birth weights, among women in sub-Saharan Africa	15 sub-Saharan African (SSA) countries	1999–2010	Retrospective cross-sectional	65,000 pregnancies	Pregnancy outcomes are indeed affected by exposure to hot days even after accounting for other individual-level characteristics
Wang et al. (2020) (38)	To investigate the association between maternal exposure to nitrogen dioxide (NO_2_) and carbon monoxide (CO) before and during pregnancy and spontaneous abortion	China	January 2014 to December 2019	Case–control study	2,445 pregnant women	Maternal exposure to NO_2_ during early pregnancy is associated with increased risk of spontaneous abortion
Bogan et al. (2021) (39)	To investigate the association between desert dust storms, particulate matter (PM10), daily temperatures, and toxemia of pregnancy and spontaneous abortion	Turkey	1 January 2009 to 31 March 2014	Retrospective cohort	6,410 total hospital admission cases were included. Of these, 6,053 were abortion cases, while 357 were cases of toxemia of pregnancy	No significant association between PM10 pollution and meteorological factors, such as maximum temperature, on the risk for adverse pregnancy outcomes was observed; however, desert dust showed a modest effect on the increased risk for toxemia of pregnancy
Xue et al. (2021) (40)	To quantify pregnancy losses attributable to ambient fine particulate matter (PM2.5) in South Asia	India, Pakistan, and Bangladesh	2000–2016	Self-compared case–control study	4,197 cases of pregnancy loss were matched with 76,282 live birth controls	A considerable proportion of the pregnancy loss burden in South Asia is attributable to exposure to ambient PM2.5. In addition, lower socioeconomic status (i.e., rural vs. urban residence) and older maternal age significantly enhanced the association between PM2.5 and pregnancy loss
Qu et al. (2021) (41)	To examine the association between extreme heat exposure (EHE) and emergency department (ED) visits and hospital admissions due to total pregnancy complications in New York State	USA	1 January 2005 to 31 December 2013	Case-crossover	1,934,918 emergency department visits and 835,465 hospital admissions due to pregnancy complications	Extreme heat exposure during pregnancy was significantly associated with increased risks of multiple subtypes of pregnancy complications, including threatened/spontaneous abortion
Liang et al. (2021) (42)	To explore the association between short-term exposure to air pollution and spontaneous abortion in Chongqing, China	China	1 January 2014 to 31 December 2018	Time series	42,334 cases of spontaneous abortion	Short-term exposure to NO_2_ may induce an increased risk of SAB outpatient visits, especially in older women and during winter seasons
Wang et al. (2021) (43)	To examine the association between maternal PM2.5 exposure and spontaneous incident pregnancy loss in China	China	2007–2010	Prospective cohort study, with sensitivity analyses including self-matched case–control	18,513 women of reproductive age	There is a significant association between maternal exposure to PM2.5 and incident spontaneous pregnancy loss
Dastoorpoor et al. (2021) (44)	To determine the relation between physiological equivalent temperature (PET) with adverse pregnancy outcomes, including stillbirth, low birth weight (LBW), preterm labor (PTL), spontaneous abortion (SA), preeclampsia, and hypertension in Ahvaz, Iran	Iran	April 2008 until March 2018	Cohort	150,766 pregnant women who visited two large referral hospitals	The results of this study showed that hot and cold thermal stress may be associated with increased risk of stillbirth and LBW
Kornfield et al. (2022) (45)	To compare pregnancy loss rates following an acute exposure to good vs. hazardous air quality from a wildfire smoke event	USA	August 20–October 31 of 2019 and 2020	Retrospective cohort	A total of 248 pregnant women, including 151 from 2019 and 97 from 2020	An acute wildfire smoke event was associated with a trend toward increased pregnancy loss in a general OB/GYN population
Zhou et al. (2022) (46)	To investigate acute and lag effects of PM2.5, PM10, NO_2_, and SO_2_ exposure on spontaneous abortion	China	1 November 2016 to 30 September 2019	Case-cross over	1,399 Pregnant women experiencing spontaneous abortion	Maternal exposure to high levels of PM2.5, PM10, NO_2_, and SO_2_ may increase the risk of spontaneous abortion through notable air quality changes, a large sample size, and precise individual exposure assessment
Khodadadi et al. (2022) (47)	To investigate the relationship between the Universal Thermal Climate Index (UTCI) and adverse pregnancy outcomes, including spontaneous abortion (SA), stillbirth, low birth weight (LBW), preterm labor (PTL), preeclampsia, and hypertension, in Ahvaz, Iran	Iran	April 2008 to March 2018		150,766 pregnant women	Overall, our results showed that heat stress measured by the UTCI index was associated with an increased risk of stillbirth, while low levels of UTCI and cold heat stress were associated with increased risk of PTL. But, no significant relation between the UTCI index with other adverse pregnancy outcomes, including abortion
Das et al. (2023) (48)	To investigate the association between ambient temperature and the incidence of miscarriage using longitudinal HDSS data from a coastal Bangladeshi population	Bangladesh	January 2012 to December 202	Cohort	13,376 pregnant women	There is a link between high ambient temperature and miscarriages
Hajdu et al. (2023) (49)	To estimate causal effects of temperature exposure on weekly spontaneous pregnancy loss rates using administrative data and to project the impacts of climate change using climate model outputs	Hungary	1984–2018	Case–control	590,872 women	Combining the estimated effects with outputs from 30 climate models suggests that climate change will increase the spontaneous pregnancy loss rate in the 21st century. The risk of pregnancy loss will be especially elevated during the summer
Xu et al. (2023) (50)	To evaluate associations between short-term exposure to six air pollutants (CO, NO_2_, SO_2_, O_3_, PM2.5, PM10) and early miscarriage to identify critical exposure windows	China	January 2017 to December 2019	Hospital-based case–control	300 (147 early miscarriage cases, 153 controls)	There is an association between short-term exposure to CO and early miscarriage
Zhao et al. (2023) (12)	To evaluate the association between ambient temperature and risk of spontaneous abortion in early pregnancy	China	January 2017 to February 2021	Case–control (matched)	1,002 cases (SAB) and 2,004 controls (total 3,006)	Temperature was an important environmental factor that affected the risk of spontaneous abortion, and exposure to high ambient temperature during early pregnancy was significantly associated with an increased risk of spontaneous abortion, especially in short-term exposure.
Wesselink et al. (2023) (24)	To estimate the effect of ambient air pollution exposure on spontaneous abortion incidence	USA and Canada	June 2013 and April 2019	Population-based cohort study	4,643 U.S. participants and 851 Canadian participants	There is little evidence for an effect of PM2.5, NO_2_, and O_3_ on SAB incidence in the United States but a moderate positive association between PM2.5 concentrations and SAB incidence in Canada
Tong et al. (2023) (13)	To examine the joint effect of ozone and temperature on pregnancy loss	USA	1989–2005	Case-crossover	247,305 pregnancy losses	Joint exposure to O_3_ and high temperature can increase the risk for pregnancy loss. The adverse effect of O_3_ is potentially modified by ambient temperature
Rekha et al. (2023) (51)	To explore the relationship between occupational heat exposures, physiological heat strain indicators, and adverse outcomes in pregnant women	India	2017–2019 and 2021–2022	Prospective Cohort	A total of 903 participants were recruited, with a final sample size of 800 pregnant women who engaged in moderate to heavy physical work	High occupational heat exposure is associated with adverse pregnancy outcomes in India
He et al. (2024) (52)	To evaluate the risk of pregnancy loss for women exposed to floods	33 developing countries	2003–2018	Matched case–control design	69,480 pregnancy losses (cases) were matched to 194,409 successful deliveries (controls)	There was a statistically significant association, reporting an odds ratio (OR) of 1.08, indicating that women exposed to floods during pregnancy are more likely to experience a pregnancy loss. This risk was particularly high for women who were either very young (<21 years) or older (>35 years)
Li et al. (2024) (53)	To examine associations between multiple air pollutants (PM2.5, PM10, SO_2_, NO_2_, CO, O_3_) and spontaneous abortion	China	2018–2019	Case–control study (1:4 matching)	289 cases of spontaneous abortion and 1,156 cases of full-term labor	Exposure to high levels of air pollutants may be a cause of increased risk of spontaneous abortion, especially in the first trimester of the last menstrual period
Moodley et al. (2024) (54)	To investigate the relationship between maternal heat exposure and miscarriage (pregnancy ending before 20 weeks of gestation)	South Africa	January 2012 and November 30, 2016	Population-based cohort study	3,477	There is a clear relationship between maternal exposure to heat during the month preceding conception and miscarriage
Wesselink et al. (2024) (24)	To examine the association of ambient heat with spontaneous abortion. The study also explored the association of cold temperatures with the risk of spontaneous abortion	USA/ Canada	June 2013– 2022	Case-cross over	1,524 participants reported spontaneous abortion. 657 spontaneous abortions occurred in the warm season and 615 in the winter season	There is an association between higher outdoor air temperature and greater spontaneous abortion incidence
Jukic et al. (2025) (55)	To examine potential associations between ambient air pollution and spontaneous miscarriage	USA	2008 and 2016	Prospective cohort	446	Ambient air pollutants may be associated with small increases in miscarriage risk. Associations were stronger among women with low vitamin D levels

PM, particulate matter; CO, carbon monooxide; NO_2_, nitrogen dioxide; SO_2_, sulfur dioxide; O_3_, ozone.

### Exposures and outcome characteristics

The included studies examined a range of climate-related exposures, including ambient air pollution (*n* = 21) (13, 24–29, 31, 32, 35–38, 40, 42, 43, 46, 50, 53, 55, 57), heat or high temperature exposure (*n* = 13) (12, 30, 33, 34, 41, 44, 47–49, 51, 54, 56, 58), floods (*n* = 1) (52), wildfire smoke (*n* = 1) (52), and dust storms and particulates matter (*n* = 1) (39). Air pollution exposure was mostly assessed using data from fixed-site monitoring stations, satellite-derived estimates, or modeled concentrations (expressed in µg/m^3^ or ppb) (13, 24–29, 31, 32, 35–38, 40, 42, 43, 46, 50, 53, 55, 57). Heat exposure and high-temperature exposures were measured using metrological data (daily mean or maximum temperature), as well as climate-based indices such as the Universal Thermal Climate Index (UTCI) and the wet-bulb globe temperature (WBGT) (12, 30, 33, 34, 41, 44, 47–49, 51, 54, 56, 58). Flood exposure was defined as whether a woman was exposed to a flood event during pregnancy (52), while wildfire smoke was assessed using the air quality index (AQI) (52). Almost all studies reported spontaneous abortion/miscarriage (Table 2). Definitions of spontaneous abortion varied across studies. Sixteen studies defined it as pregnancy loss occurring before 20 weeks (13, 24, 28, 32, 37–40, 42, 45, 46, 51, 52, 54, 55, 57), five studies applied a 28-week cutoff (34, 44, 48, 49, 53), and one study used a 25-week threshold (29). Other studies used shorter gestational windows, such as ≤12 weeks (12, 33, 50, 56) or ≤14 weeks (25–27, 35, 36, 43). Three studies broadly described abortion as pregnancy loss before fetal viability without specifying a gestational cutoff (30, 31, 47). Outcome ascertainment was majorly based on medical records (*n* = 21) (12, 13, 24–26, 28, 29, 34–36, 38, 39, 41, 42, 44, 46, 47, 50, 53, 54, 56), while nine studies relied on self-reported data (*n* = 9) (24, 27, 30–32, 43, 48, 55). Two studies ascertained outcome using both medical records and self-reported data (45, 51), while others relied on Demographic and Health Survey (DHS) data and other registries (33, 37, 40, 49, 52, 57). Most studies (*n* = 29) adjusted for maternal and environmental confounders, such as maternal age, parity, history of previous miscarriage, and relevant meteorological factors. However, eight studies did not specify whether confounders were adjusted for in their analyses (24, 28, 39, 45, 46, 49, 56) (Table 2).

**Table 2:** Climate-related exposures and abortion outcome definitions of included studies (*n* = 37).

Author, year	Exposure (climate change indicator)	Exposure measurement method	Adjusted confounding factors	Definition of outcome	Outcome measurement methods
Bianchi-Demicheli et al. (2001) (24)	Atmospheric pressure, temperature, humidity, and global radiation	Atmospheric pressure was measured using a high-precision barometer. Temperature and humidity were recorded using a ventilated thermo-hygrometer. Global solar radiation was assessed by a pyranometer. The change in atmospheric pressure was calculated by subtracting the mean pressure on a given day from that on the subsequent day	Not specified	Spontaneous abortion at 8.6 ± 2 weeks	Medical records
Hou et al. (2013) (25)	Ambient air pollution	Daily average concentrations in micrograms per cubic meter (μg/m^3^) of sulfur dioxide (SO_2_), nitrogen dioxide (NO_2_), total suspended particulate (TSP), and particulate matter with aerodynamic diameters ≤10 µm (PM10)	Maternal age, gravidity, parity, and history of previous miscarriage	Spontaneous abortion at 14 weeks of pregnancy	Medical records
Thimonier et al. (2014) (26)	Ambient air pollution	Average of pollutant concentration	Maternal age, father age, cigarette smoke exposure, occupation, prepregnancy BMI, other demographic parameters, history of previous abortion, consanguinity with spouse, and educational status	Spontaneous abortion at 14 weeks of pregnancy	Medical records
Moridi et al. (2014) (27)	Ambient air pollutants (CO, NO_2_, SO_2_, O_3_ and PM10)	Pollutant concentrations were collected hourly at 29 fixed-site stations. Units were parts per million (ppm) for CO, parts per billion (ppb) for SO_2_, NO_2_, and O_3_, and micrograms per cubic meter (μg/m^3^) for PM10	Maternal age, father age, gravidity, duration from last delivery, history of previous abortion, prepregnancy BMI, occupation, educational status, secondhand smoke exposure, socioeconomic status, consanguinity with spouse, and duration of residence	Spontaneous abortion before 14 weeks of gestation	Self-reported
Enkhmaa et al. (2014) (28)	Ambient air pollutants, including ozone (O_3_), sulfur dioxide (SO_2_), nitrogen dioxide (NO_2_), carbon monoxide (CO), PM	Monthly average levels of pollutants were measured. The units are micrograms per cubic meter (μg/m^3^) for PM10 and PM2.5, milligrams per cubic meter (mg/m^3^) for CO, and parts per billion (ppb) for SO_2_, NO_2_, and O_3_	Not specified	Spontaneous abortion before 20 weeks of gestational age	Medical records
Di Ciaula et al. (2015) (29)	Air pollutants (PM10, ozone, and, NO_2_)	Average monthly air levels in micrograms per cubic meter (μg/m^3^)	Air temperature and humidity	Spontaneous abortion within 25 weeks of gestation	Medical records
Asamoah et al. (2017) (30)	Maternal heat exposure	Levels of the wet-bulb globe temperature (WBGT). WBGT is a heat index based on temperature, humidity, heat radiation, and air movement	Maternal age at the end of pregnancy and total number of pregnancies (gravidity)	Spontaneous abortion, timing not specified	Self-reported
Ha et al. (2018) (31)	Air pollution (ozone, PM2.5, PM10, NO_2_, SO_2_, CO, and particulate constituents including sulfate, nitrate, ammonium, organic carbon, elemental carbon)	Average daily pollutant concentrations (µg/m^3^)	Season, study site, maternal age, maternal race, parity, maternal education, household income, caffeine intake, BMI, multivitamin intake, and maternal and paternal serum cotinine levels	Spontaneous abortion, timing not specified	Self-reported
Gaskins et al. (2019) (32)	Ambient air pollution	PM concentrations in μg/m^3^; predicted monthly ambient PM concentrations from nationwide spatiotemporal models	Age, smoking status, year of pregnancy, BMI, history of infertility, multivitamin use, marital status, race, region, census tract median income, census tract median home value	Spontaneous abortion at 20 weeks of gestation	Self-reported
Xue et al. (2019) (33)	Ambient fine particulate matter	PM2.5 concentration in μg/m^3^; monthly averages in 0.1° × 0.1° grids	Maternal age during the year of delivery, BMI, smoking, anemia, and employment status	Miscarriage/Spontaneous abortion at 20 weeks of gestation	Demographic and Health Survey
Sun et al. (2019) (34)	High temperature	Daily meteorological data (daily mean temperature and precipitation)	Maternal age, race, and average precipitation	Spontaneous abortion at 28 weeks of gestation	Medical records
Zhang et al. (2019) (35)	Ambient air pollutants (particulate matter (PM2.5), (SO_2_), ozone (O_3_), and carbon monoxide (CO)	Milligrams per cubic meter (mg/m^3^)	Sociodemographic characteristics, spatial autocorrelation, ambient temperature, maternal age, and occupation	Missed abortion in the 14 weeks of gestation	Medical records
Zhang et al. (2019) (36)	Ambient fine particulate matter	Micrograms per cubic meter (µg/m^3^)	Body mass index (BMI), parity, maternal education, economic status, interior renovation, occupational exposure, alcohol consumption, and active and passive smoking during pregnancy	Early pregnancy loss (spontaneous abortion) before 13 weeks of gestation	Medical records
Leiser et al. (2019) (37)	Ambient air pollutants (NO_2_), fine particulate matter (PM2.5), and ozone (O_3_)	Average daily concentrations of NO_2_ in ppb, PM2.5 in µg/m^3^, and O_3_ in ppb	Daily average temperature	Spontaneous pregnancy loss before 20 weeks of gestation	Data Warehouse
Davenport et al. (2020) (33)	Temperature and precipitation	Daily maximum temperature and monthly rainfall were measured. The temperature data were in degree Fahrenheit, and the study counted “hot days” within specific bins (95°F–99°F, 100°F–104°F, and 105°F+)	Location, season, and year. They also adjusted for the number of children under 5, maternal age, educational attainment, household wealth (floor material), and place of residence (urban/rural)	Miscarriage within the first 12 weeks of gestation	Survey data
Wang et al. (2020) (38)	Air pollution (NO_2_, CO)	Daily concentrations of NO_2_ and CO were used. The units are micrograms per cubic meter (μg/m^3^) for NO_2_ and milligrams per cubic meter (mg/m^3^) for CO	Gestational age, maternal age, body mass index (BMI), maternal parity, marital status, the use of assisted reproduction technology temperature, and relative humidity	Spontaneous abortion before 20 weeks of gestation	Medical records
Bogan et al. (2021) (39)	Desert dust storms, particulate matter with a diameter ≤10 μm (PM10), and daily temperatures	Daily PM10 levels (μg/m^3^), daily temperature ranges (°C), mean temperature, humidity, pressure, and wind speed were collected	Not specified	Spontaneous abortion before 20 weeks of gestation	Medical records
Xue et al. (2021) (40)	Ambient fine particulate matter (PM2.5)	Milligrams per cubic meter (mg/m^3^)	Maternal age, non-linear terms for temperature and humidity, seasonal variation, and long-term trends	Pregnancy loss (miscarriage) before 20 weeks gestation	Demographic and Health Surveys
Qu et al. (2021) (41)	Ambient extreme heat exposure	Daily mean temperature was above the 90th percentile of the monthly mean temperature	Air pressure, wind speed, rainfall, relative humidity, ambient fine particulate matter (PM2.5), ozone concentrations	Threatened or spontaneous abortion before 28 weeks	Medical record
Liang et al. (2021) (42)	Ambient nitrogen dioxide	Daily concentrations of NO_2_ in µg/m^3^	Meteorological conditions (mean temperature and relative humidity) and day of the week	Spontaneous abortion before 20 weeks of gestation	Medical records
Wang et al. (2021) (43)	Ambient fine particulate matter (PM2.5)	PM2.5 concentration (µg/m^3^), average across pregnancy, and county-level estimates	Temporal trend, maternal age, pregnancy intentionality, residence (urban/rural), education, household income, employment status	Spontaneous pregnancy loss (miscarriage) occurring at >13 weeks; induced abortion also recorded but treated as censoring	Self-reported
Dastoorpoor et al. (2021) (44)	Physiological equivalent temperature	PET is a thermal index. The data required to calculate the PET index include air temperature, relative humidity, wind speed, cloudiness, vapor pressure, radiation temperature, and global radiation	Air pollutants (NO_2_, SO_2_ and PM10)	Spontaneous abortion before 28 weeks	Medical records
Kornfield et al. (2022) (45)	Wildfire smoke	Air quality index (AQI)	Not specified	Pregnancy loss before 20 weeks of gestation	Self-reported and medical records
Zhou et al. (2022) (12)	Ambient air pollution	Daily average pollutant concentrations (µg/m^3^ or mg/m^3^ for CO; ppb for gases)	Not specified	Spontaneous abortion, <20 weeks of gestation	Medical records
Khodadadi et al. (2022) (47)	Universal Thermal Climate Index (UTCI)	Temperature in °C	Time trend, air pollutants (NO_2_, SO_2_, and PM 10), and weekdays	Spontaneous abortion (timing not specified)	Medical records
Das et al. (2023) (48)	Ambient temperature	Daily average temperature (°C)	Mother's age at outcome, pregnancy order, education level, and distance from the sea	Spontaneous abortion at 28 weeks of gestation	Self-reported
Hajdu et al. (2023) (49)	Daily mean temperature	Mean daily temperature (°C)	Not specified	Spontaneous abortion, 20–28 completed weeks of gestation	Administrative registries
Xu et al. (2023) (50)	Air pollution (CO, SO_2_, NO_2_, O_3_, PM2.5, PM10)	Daily average concentrations (µg/m^3^ or mg/m^3^ for CO) from municipal monitoring stations	Maternal age, gestational age, gravidity, parity, prior abortion history, LMP season, average temperature, relative humidity, and model variants	Early miscarriage at 8–10 weeks of gestation	Medical records
Zhao et al. (2023) (12)	Ambient temperature	Daily mean temperature (°C) from Meteorological Data Sharing Service	Maternal age, gestational age, and history of previous miscarriages	Spontaneous abortion before 12 weeks of gestation	Medical records
Wesselink et al. (2023) (24)	Ambient air pollution	Average ambient concentrations of PM2.5 in µg/m^3^ and NO_2_ and O_3_	Not specified	Spontaneous abortion at 20 weeks of gestation	Self-reported
Tong et al. (2023) (13)	Ozone (O_3_) and temperature	O_3_ was measured as the daily maximum 8-h average in μg/m^3^. Temperature was the 24-h average in °C	Relative humidity (RH), height of the planetary boundary layer (PBL), and holidays	Miscarriage before 20 weeks of gestation	Medical records
Rekha et al. (2023) (51)	Heat exposure	The exposure was measured using the WBGT in °C	Age, education, consanguinity, socioeconomic status (SES), occupation, BMI, and hemoglobin level	Miscarriage before 20 weeks of gestation	Self-reported and medical records
He et al. (2024) (52)	Floods	The exposure was measured by whether a woman was exposed to a flood event during her pregnancy. The average flood exposure was 25.6 days	Maternal age, gestational month, preconception exposure, socioeconomic factors, and living conditions	Miscarriage before 20 weeks of gestation	Demographic and Health Surveys
Li et al. (2024) (53)	Air pollutants (PM2.5, PM10, SO_2_, NO_2_, CO, O_3_)	Daily concentrations (µg/m^3^ for PM/NO_2_/SO_2_/O_3_, mg/m^3^)	Age, gestation, delivery, dissection, season of last menstruation, menstrual pattern, pregnancy complications, pregnancy comorbidities, and hypertension during pregnancy factors	Spontaneous abortion at 28 weeks of gestation	Medical records
Moodley et al. (2024) (54)	Heat exposure	Daily temperature of >26.6°C	Survey year, maternal age, socioeconomic status, education level, distance to the nearest healthcare clinic, HIV, and tuberculosis	Spontaneous abortion at 20 weeks of gestation	Medical records
Wesselink et al. (2024) (24)	Ambient outdoor air temperature	Daily maximum outdoor air temperatures were measured	Not specified	Spontaneous abortion before 20 weeks of gestation	Self-reported
Jukic et al. (2025) (55)	Ambient air pollution, specifically PM10, PM2.5, CO, NO, NO_2_, SO_2_, and O_3_	Concentrations were estimated at a 12-km × 12-km grid resolution. Gaseous pollutants were measured in ppb, and particulate matter was measured in µg/m^3^. Both average and single-day peak concentrations were assessed	Age, partner age, parity, education, body mass index (BMI), season, race/ethnicity, partner race/ethnicity, median household income, and daily average land surface temperature	Spontaneous miscarriage before 20 weeks of gestation	Self-reported

ppb, parts per billion; PM, particulate matter; CO, carbon monooxide; NO_2_, nitrogen dioxide; SO_2_, sulfur dioxide; O_3_, ozone.

### Quality assessment of included studies

The methodological quality of the 37 included studies is summarized in Supplementary File 3, with each item depicted. Based on the Newcastle–Ottawa Scale (NOS), 84% of the studies were deemed high quality, while the remaining 16% were classified as moderate quality.

### Air pollution and spontaneous abortion

**Figure 3:**
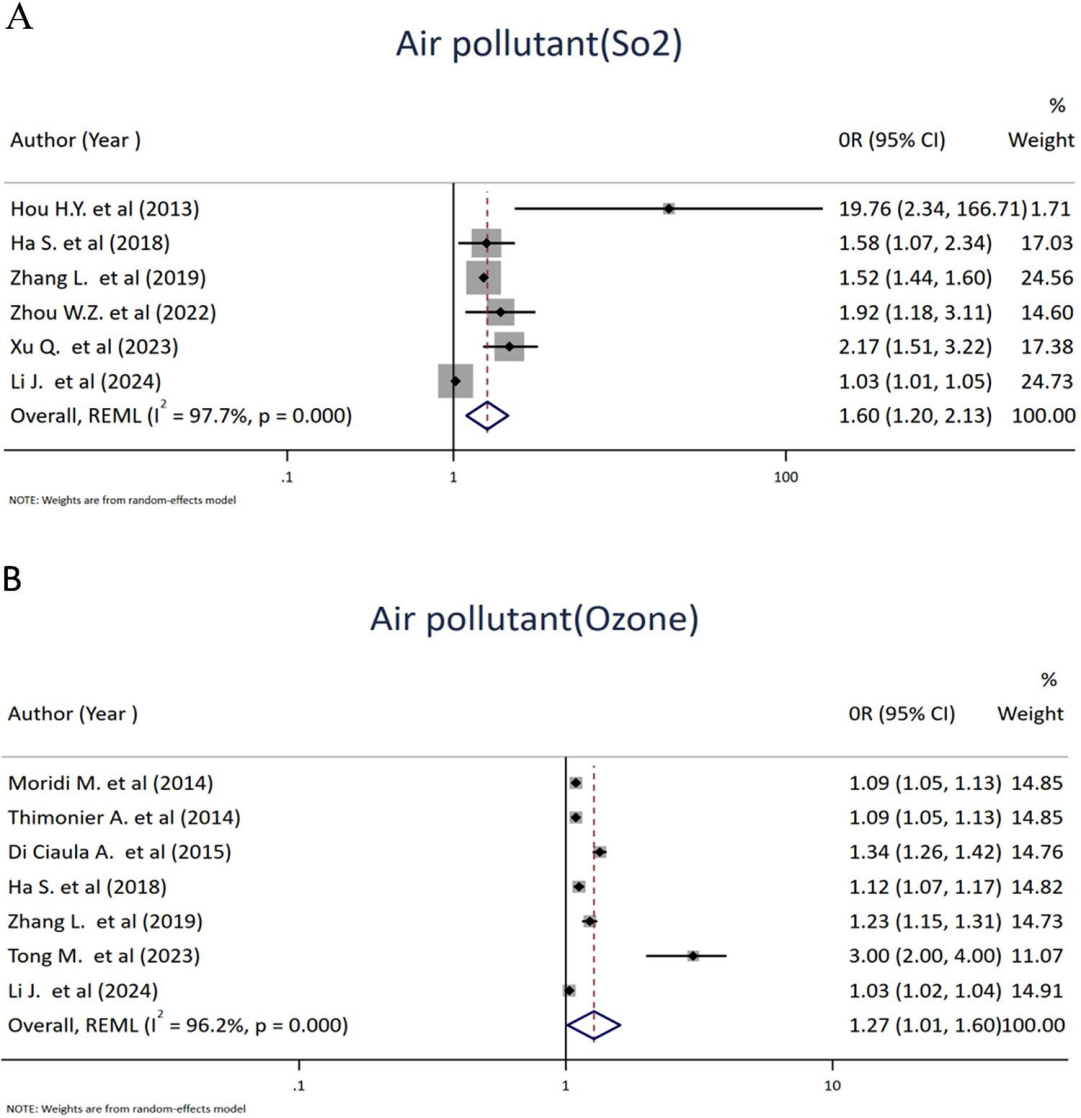
Forest plot of the association between sulfur dioxide (SO_2_) **(A)** and ozone **(B)** and risk of abortion. Each diamond (dot) represents the study-specific odds ratio (OR), with the size of the diamond proportional to the study weight in the meta-analysis. The horizontal line through each diamond indicates the 95% confidence interval (CI). The vertical solid line at OR = 1 represents the line of no effect. The dashed vertical line represents the pooled effect estimate from the random-effects model. The diamond at the bottom represents the pooled OR, with its width corresponding to the 95% CI. Heterogeneity is expressed using the *I*^2^ statistic.

A total of 21 studies reported the impact of air pollution on abortion. The studies included in this review evaluated associations between five major air pollutants and abortion, including sulfur dioxide (SO_2_), ozone (O_3_), carbon monoxide (CO), nitrogen dioxide (NO_2_), and particulate matter (PM). Six studies that reported effect sizes were included to investigate the association between ambient air pollutant sulfur dioxide (SO_2_) and abortion. The pooled results revealed a significant positive association between sulfur dioxide (SO_2_) exposure and spontaneous abortion among women, with an OR value of 1.60 (95% CI: 1.20, 2.13; *I*^2^ = 97.7%, *p* = <0.001). Pooled analysis of seven studies on the association between ozone (O_3_) exposure during pregnancy and spontaneous abortion showed a positive association, with an OR value of 1.27 (95% CI: 1.01, 1.60; *I*^2^ = 96.2%, *p* = <0.001) (Figures 3A,B).

**Figure 4:**
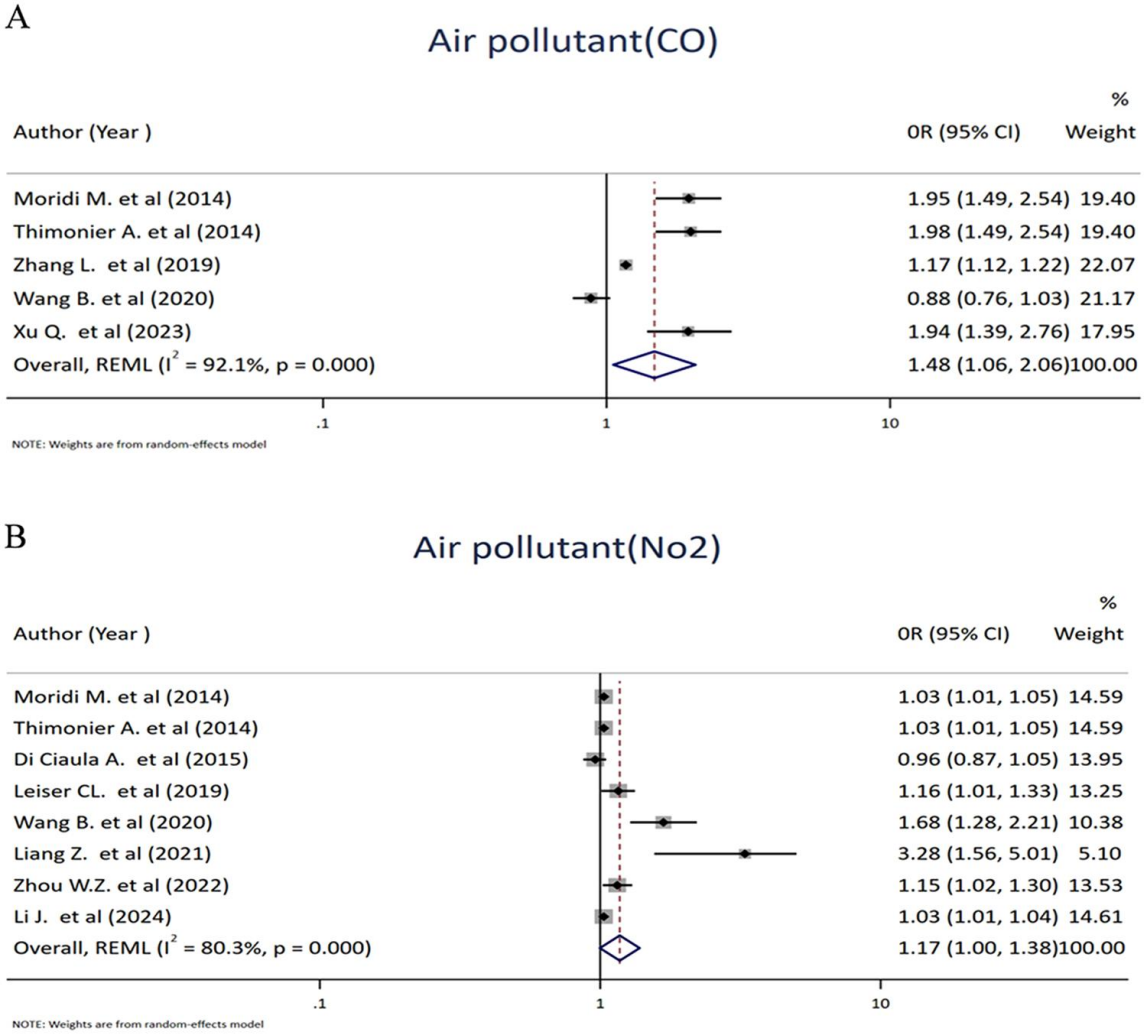
Forest plot of the association between carbon monoxide (CO) **(A)** and nitrogen dioxide (NO_2_) **(B)** and risk of abortion. Each diamond (dot) represents the study-specific odds ratio (OR), with the size of the diamond proportional to the study weight in the meta-analysis. The horizontal line through each diamond indicates the 95% confidence interval (CI). The vertical solid line at OR = 1 represents the line of no effect. The dashed vertical line represents the pooled effect estimate from the random-effects model. The diamond at the bottom represents the pooled OR, with its width corresponding to the 95% CI. Heterogeneity is expressed using the I^2^ statistic.

Five studies that reported the effects of carbon monoxide exposure were pooled to assess their impacts on spontaneous abortion. Accordingly, the odds of spontaneous abortion among women were increased by 48% for women exposed to carbon monoxide, with an OR value of 1.48 (95% CI: 1.06, 2.06; *I*^2^ = 92.1%). Furthermore, eight studies investigated the relationship between nitrogen dioxide (NO_2_) exposure and the occurrence of spontaneous abortion. The pooled estimate suggested that women exposed to NO_2_ had a 17% higher odds of spontaneous abortion than those not exposed (Figures 4A,B).

**Figure 5:**
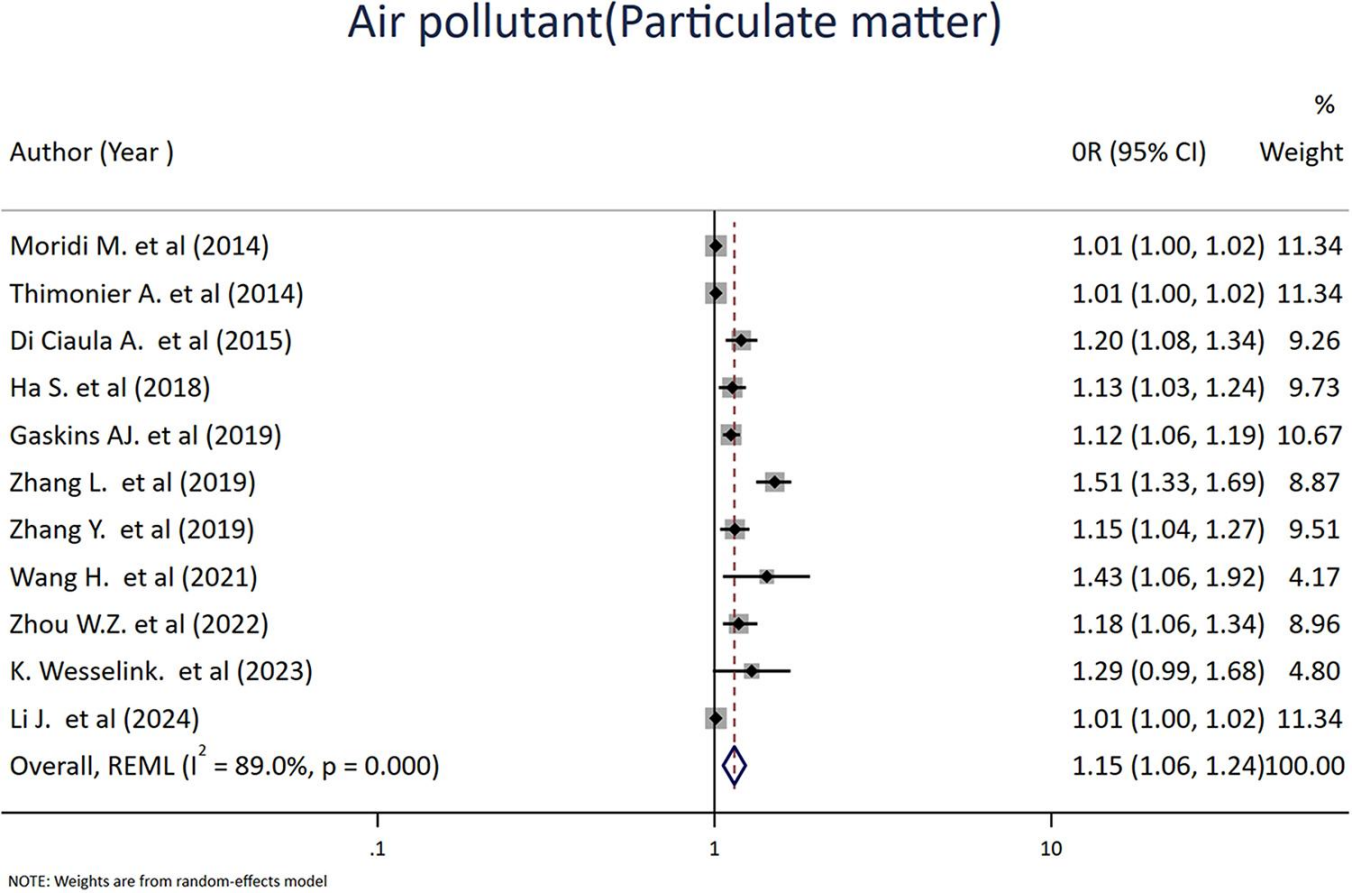
Forest plot of the association between particulate matter (PM) and risk of abortion. Each diamond (dot) represents the study-specific odds ratio (OR), with the size of the diamond proportional to the study weight in the meta-analysis. The horizontal line through each diamond indicates the 95% confidence interval (CI). The vertical solid line at OR = 1 represents the line of no effect. The dashed vertical line represents the pooled effect estimate from the random-effects model. The diamond at the bottom represents the pooled OR, with its width corresponding to the 95% CI. Heterogeneity is expressed using the *I*^2^ statistic.

Similarly, 11 studies assessed the association between maternal exposure to particulate matter (PM) and spontaneous abortion. Particulate matter consists of a complex mixture of solid and liquid particles suspended in the air and is commonly classified by aerodynamic diameter as PM2.5 (≤2.5 µm) and PM10 (≤10 µm). The pooled results revealed a significant positive association between particulate matter exposure and spontaneous abortion, with an OR value of 1.15 (95% CI: 1.06–1.24; I^2^ = 89.0%, *p* = <0.001) (Figure 5).

### Ambient air temperature/heat exposure and spontaneous abortion

**Figure 6:**
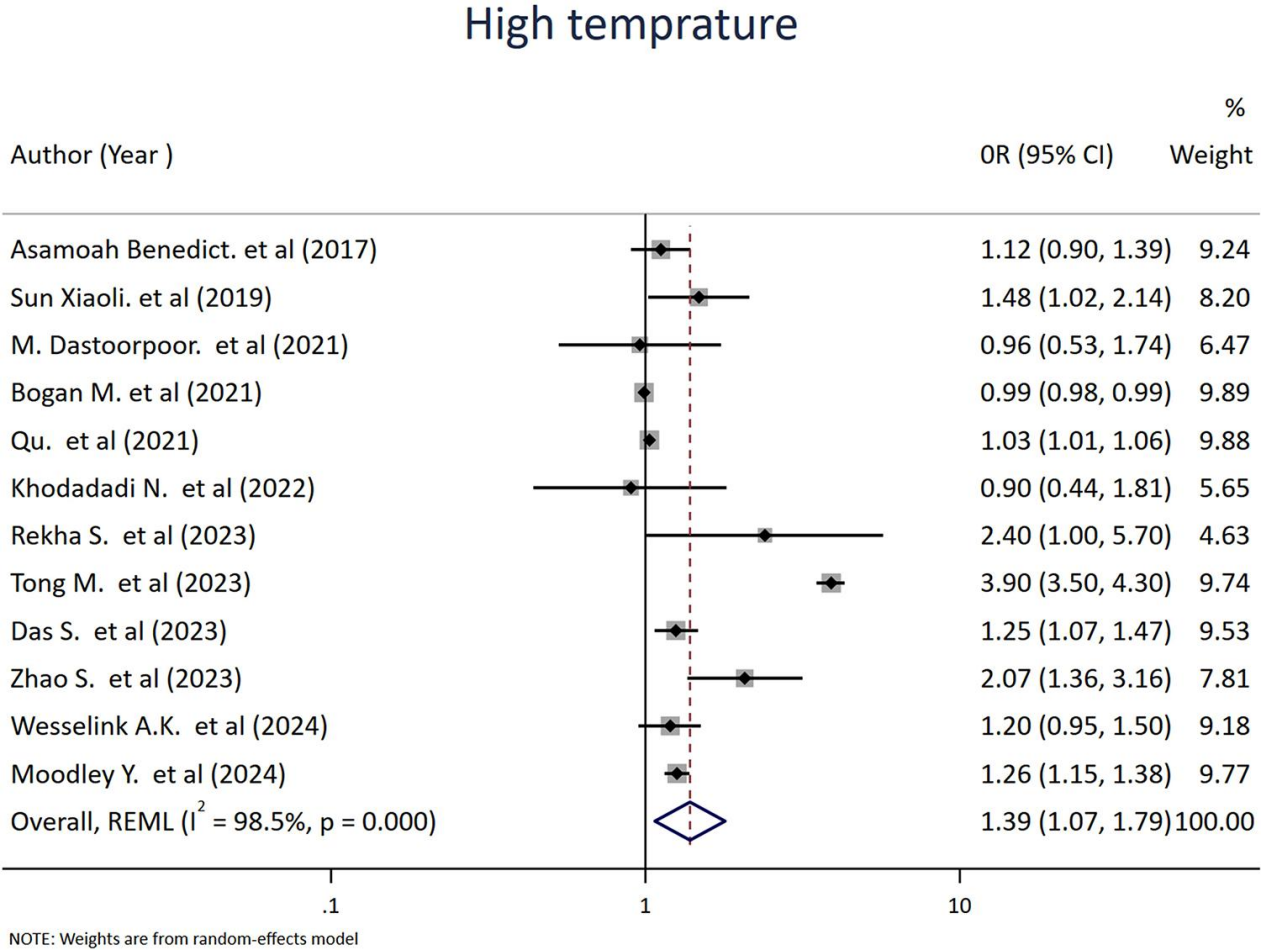
Forest plot of the association between high temperature and risk of abortion. Each diamond (dot) represents the study-specific odds ratio (OR), with the size of the diamond proportional to the study weight in the meta-analysis. The horizontal line through each diamond indicates the 95% confidence interval (CI). The vertical solid line at OR = 1 represents the line of no effect. The dashed vertical line represents the pooled effect estimate from the random-effects model. The diamond at the bottom represents the pooled OR, with its width corresponding to the 95% CI. Heterogeneity is expressed using the *I*^2^ statistic.

We identified 12 studies that evaluated the impact of ambient air temperature, including exposure to extreme heat during pregnancy, on abortion. Accordingly, the pooled estimates showed that high-temperature exposure was significantly associated with an increased risk of spontaneous abortion, with an OR of 1.15 (95% CI: 1.07–1.79; *I*^2^ = 89.0%, *p* ≤ 0.001) (Figure 6).

### Extreme events (wildfires and floods) and spontaneous abortion

There is limited data on the impact of wildfires on abortion. Among the included studies, only one study conducted in the United States (45) assessed the impact of wildfire smoke on spontaneous abortion. The study concluded that acute wildfire smoke events were associated with a trend toward increased pregnancy loss (abortion). Similarly, only one study evaluated the association between flood exposure and abortion (52). This study revealed a statistically significant association, with an OR of 1.08, indicating that women exposed to floods during pregnancy were more likely to experience pregnancy loss. It also noted that the risk was higher among women dependent on surface water and those with lower income or education levels. According to the study, the odds of pregnancy loss were significantly higher for women who experienced prolonged flood exposure (>16 days; OR: 2.00, 95% CI: 1.83–2.18) and those exposed to flooding on multiple occasions (≥2 events; OR: 1.78, 95% CI: 1.69–1.88).

### Subgroup analysis

**Figure 7:**
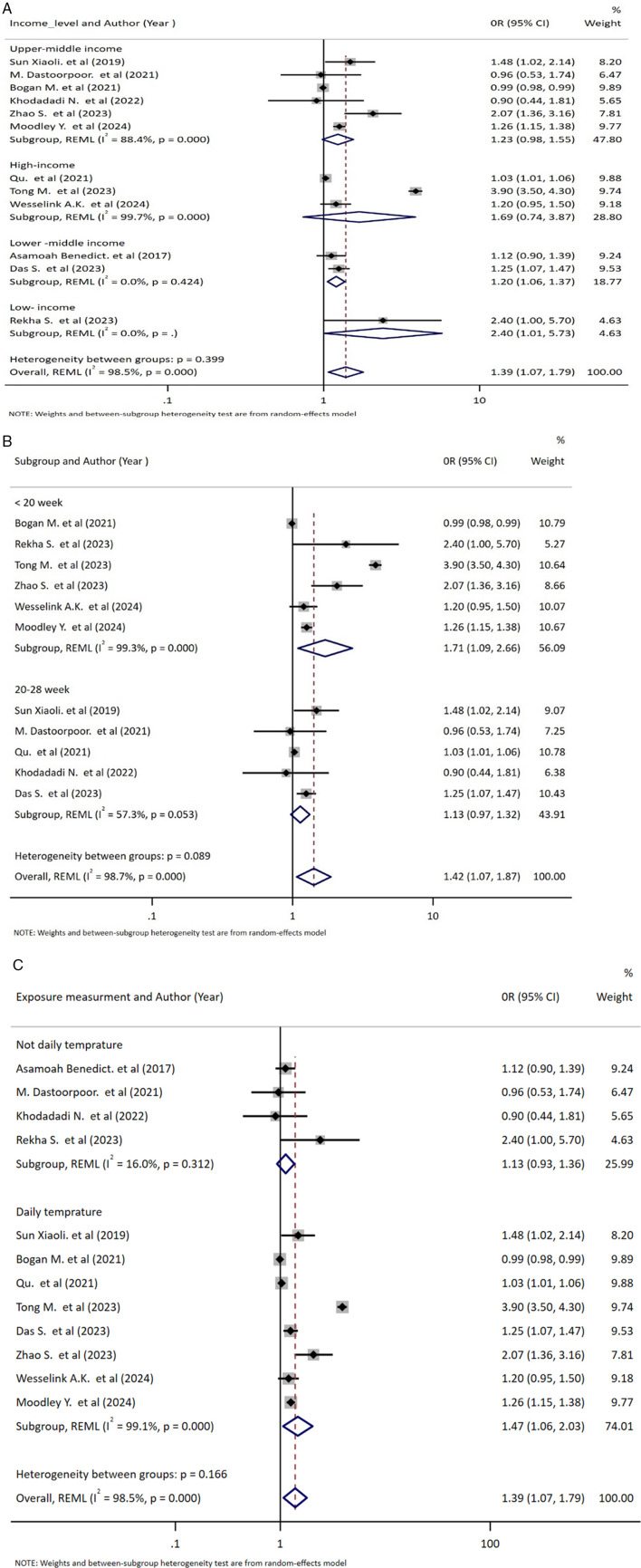
Forest plot showing subgroup analysis of the association between high temperature and risk of abortion based on country income level **(A)**, gestational age **(B)**, and exposure measurement method **(C)**. Each diamond (dot) represents the study-specific odds ratio (OR), with the size of the diamond proportional to the study weight in the meta-analysis. The horizontal line through each diamond indicates the 95% confidence interval (CI). The vertical solid line at OR = 1 represents the line of no effect. The dashed vertical line represents the pooled effect estimate from the random-effects model. The diamond at the bottom represents the pooled OR, with its width corresponding to the 95% CI. Heterogeneity is expressed using the *I*^2^ statistic.

In this review, to explore possible sources of heterogeneity, subgroup analyses were performed for high ambient temperature exposure based on country income level, gestational window, and exposure measurement method. The income-level analysis showed that high-temperature exposure increased the risk of spontaneous abortion in low- and lower-middle-income countries, with the highest risk observed in low-income settings. Eleven studies were included in the subgroup analysis stratified by the definition of abortion or gestational age. The result showed that abortion defined as occurring before 28 weeks showed reduced heterogeneity (*I*^2^ = 57.3%, *P* = 0.053), and the association was not statistically significant (OR: 1.13, 95% CI: 0.97, 1.32). In contrast, spontaneous abortion defined as occurring before 20 weeks showed a statistically significant association with high-temperature exposure (OR: 1.42; 95% CI: 1.07, 1.87). When studies with different measurement methods were combined, high heterogeneity was observed among studies using daily temperature measurements (*I*^2^ = 99.1%, *P* = 0.053), while heterogeneity was insignificant among studies using other measurement methods (*I*^2^ = 16.0%, *P* = 0.321). Accordingly, the pooled estimate for studies using daily temperature measurements was statistically significant (OR: 1.47; 95% CI: 1.06, 2.03), while the pooled estimate for other measurement methods was not statistically significant (OR: 1.13; 95% CI: 0.93, 1.36) (Figures 7A–C).

### Sensitivity analyses

Sensitivity analyses were conducted using a leave-one-out diagnostic approach for studies examining high ambient temperature and particulate matter exposure, indicating that no single study had a substantial influence on the overall pooled estimates. Across all iterations, the pooled odds ratios remained stable and statistically significant (Supplementary File 4).

### Publication bias

Our analysis involved a funnel plot along with Egger's test to assess publication bias for high ambient temperature and particulate matter exposure; hence, they deemed a sufficient number of studies. Accordingly, the test yielded *p*-values of 0.09 and 0.06, respectively, suggesting no statistically significant publication bias or small-study effects in all pooled analyses.

## Discussion

This systematic review and meta-analysis synthesized evidence from 37 studies conducted across Asia, Africa, Europe, and North America to examine the relationship between climate change-related exposures and abortion. Our findings indicate that multiple climate-related exposures, including air pollution, heat exposure, floods, and wildfire smoke, are significantly associated with an increased risk of abortion. Pooled results revealed that exposure to sulfur dioxide, ozone, carbon monoxide, nitrogen dioxide, and particulate matter increased the odds of abortion. Similarly, maternal exposure to elevated temperatures and heat waves was linked to an increased risk of abortion. Although fewer studies assessed floods and wildfire smoke, the available evidence suggests a positive association with abortion.

Air pollution emerged as the most studied exposure, with strong and consistent associations observed across pollutants such as SO_2_, NO_2_, CO, O_3_, and particulate matter (PM2.5/PM10), all of which were significantly associated with an increased risk of spontaneous abortion. This finding is consistent with an earlier review linking air pollutants to adverse pregnancy outcomes, including abortion (59–61). These associations might be explained by several mechanisms. In general, air pollutants can trigger oxidative stress, systemic inflammation, hormonal disruption, and impaired placental vascular development, all of which may interfere with implantation and early gestation (62, 63). More specifically, sulfur dioxide may exert harmful effects by its rapid conversion to sulfite and bisulfite ions, which can cause oxidative damage to maternal and placental tissues (64). The stronger associations observed in studies focusing on first-trimester exposure support these pathways, given the critical role of early placentation.

In contrast, the association between carbon monoxide and abortion is explained by its unique ability to bind hemoglobin with high affinity, which reduces oxygen transport and causes maternal and fetal hypoxia (65). Importantly, studies have reported stronger effects of O_3_ on hotter days, suggesting an interaction between air pollution and ambient temperature. This aligns with the reality of climate change, as rising temperatures enhance ozone formation (66). Our pooled analysis demonstrated a modest but significant association between NO_2_ exposure and abortion. The association might also be explained by the strong oxidative capacity of NO_2_, which can damage airway epithelium, induce endothelial dysfunction, and reduce utero-placental blood flow (67). More specifically, particulate matter can penetrate the bloodstream and placenta, carrying toxic components that can directly damage trophoblasts (68).

The observed association between maternal exposure to high ambient temperature and spontaneous abortion aligns with findings from prior reviews on climate extremes and pregnancy outcomes (69). Although the exact biological mechanisms linking high temperature exposure and abortion are not fully understood, several pathways have been proposed. However, the association might be explained by heat-induced dehydration, reduced plasma volume, and maternal hyperthermia, all of which can impair utero-placental blood flow. In addition, heat may induce oxidative stress and disrupt hormonal regulation, especially progesterone balance, which is essential for maintaining pregnancy (70). Our subgroup analyses indicate that risks are heightened during early gestation and in low-income countries, with a substantial reduction in heterogeneity, highlighting both physiological and socioeconomic vulnerability. This discrepancy may be attributed to the heightened sensitivity of early pregnancy (a critical period of embryonic development) to environmental exposures. Subgroup analyses revealed stronger associations in low-income countries, where health systems are often fragile, antenatal care coverage is limited, and adaptive capacity to climate stressors remains constrained.

The subgroup analysis further indicated that the association between high ambient temperature and abortion varied according to the exposure assessment method. Studies using daily temperature measurements showed a stronger and statistically significant association, although with substantial heterogeneity, likely reflecting differences in heat definitions, lag periods, climatic settings, and population characteristics but also suggesting that daily metrics may better capture acute heat stress during sensitive periods of pregnancy. In contrast, studies using other temperature measurement approaches demonstrated low heterogeneity and a non-significant association, which may indicate that aggregated or averaged temperature measures dilute short-term extremes and underestimate their potential impact. Overall, these findings highlight the importance of high-resolution exposure assessment in detecting heat-related reproductive risks and underscore the need for standardized temperature metrics in future studies.

Although fewer studies examined the effects of floods, our synthesis suggested a positive association between flood exposure and spontaneous abortion, with stronger risks observed for prolonged flood events. Mechanistically, floods can disrupt access to antenatal care, increase the risk of infectious disease, exacerbate food insecurity, and trigger acute and chronic stress responses, all of which can contribute to pregnancy loss (71). The study also revealed greater vulnerability among adolescents and older mothers. These findings highlight the need for disaster preparedness plans that explicitly include reproductive health, such as the prepositioning of maternal health supplies, maintenance of mobile antenatal services, and provision of psychosocial support for pregnant women in flood-affected regions.

In addition, according to the study, wildfire smoke exposure increases the risk of abortion, a finding consistent with prior narrative reviews linking wildfire-related air pollutants and toxic gases to adverse pregnancy outcomes (72). Mechanistically, wildfire smoke contains fine particulate matter, carbon monoxide, and volatile organic compounds that collectively induce systemic oxidative stress, inflammation, and placental hypoxia, pathways that closely resemble those observed for ambient air pollution. While data remain sparse, the plausibility of this association is strong, and the increasing frequency and intensity of wildfires in the context of climate change make this an urgent research priority.

Taken together, our findings resonate with international calls from the WHO, UNFPA, and the IPCC to integrate reproductive health into climate adaptation strategies. Abortion risk, as a climate-sensitive outcome, highlights how environmental change intersects with gender inequities. This review provides evidence to inform targeted interventions, such as pregnancy-specific heat–health action plans, improved air quality monitoring, continuity of antenatal care during disasters, and greater protection for women working in heat-exposed occupations.

## Strengths and limitations

This systematic review and meta-analysis has several notable strengths. First, it is the most comprehensive review to date, specifically addressing the relationship between climate change-related stressors and spontaneous abortion. It summarizes findings from 37 studies conducted across four continents. Nevertheless, several limitations must be acknowledged. Considerable heterogeneity was observed among the included studies. Although subgroup analyses were performed for two exposures with sufficient numbers of studies, heterogeneity persists for other associations. The geographical distribution of studies was uneven, with most findings from Asia and North America and limited data from Africa and Latin America—regions that are particularly vulnerable to climate impacts. In addition, all included studies were observational in design, limiting the ability to establish causal relationships or explain the underlying mechanisms linking ambient temperature to spontaneous abortion.

Another limitation concerns the classification of outcomes in the primary studies. Although this review included only studies reporting spontaneous abortion (miscarriage), distinguishing spontaneous from induced abortion can be challenging in some settings due to legal restrictions, stigma, and reporting practices. Many included studies relied on medical records or routine data sources, in which induced abortions may be underreported or misclassified as spontaneous pregnancy loss. Because this review synthesized evidence across diverse legal and sociocultural contexts, some degree of residual misclassification may persist.

## Conclusion

This systematic review and meta-analysis provides evidence that climate change-related exposures, including air pollution, extreme heat, flooding, and wildfires, are significantly associated with an increased risk of abortion, particularly spontaneous abortion. These results show that abortion is not only a consequence of reproductive health but also an indicator of vulnerability to climate change. Addressing this issue requires the urgent integration of reproductive health into climate change adaptation strategies, health system strengthening efforts, and the global justice agenda. Future research should prioritize underrepresented regions, including low- and middle-income countries, and examine emerging climate-related threats to ensure that women's reproductive health and rights are protected in the era of accelerating climate change.

## Practical implications

The findings of this study have important implications for policy, health systems, and practice. First, reproductive health needs to be explicitly included in climate change adaptation and mitigation strategies. National adaptation plans and climate resilience frameworks should include pregnancy-specific measures for pregnant women, improved monitoring of pregnancy outcomes during climate events, and emergency preparedness measures that ensure continuity of antenatal care and reproductive health services.

Second, health systems, particularly in low- and middle-income countries, must be better able to monitor, prevent, and respond to climate-related reproductive risks. This includes investing in climate-resilient health infrastructure, expanding mobile and community-based services during extreme events, and developing early warning systems to alert health workers and pregnant women to harmful exposures, such as heat waves, flooding, and air pollution. Third, policymakers should prioritize measures to promote gender equality, as women are disproportionately at risk in resource-constrained areas. Finally, the findings emphasize the need for cross-sectoral collaboration. Environmental agencies, disaster management agencies, and ministries of health need to coordinate their efforts to reduce exposure, while reproductive health programs should incorporate environmental risk education and counseling into routine antenatal care.

## Data Availability

The datasets presented in this study can be found in online repositories. The names of the repository/repositories and accession number(s) can be found in the article/Supplementary Material.

## Supplementary material

SUPPLEMENTARY FILE 1 Search strategies.

SUPPLEMENTARY FILE 2 Reasons for exclusion of studies.

SUPPLEMENTARY FILE 3 Quality assessment of the included studies.

SUPPLEMENTARY FILE 4 Sensitivity analysis for high ambient temperature and particulate matter exposure.
