# Supplementary material for: Impact of climate change on spontaneous abortion: a systematic review and meta-analysis
Source: Front Glob Womens Health. 2026 Mar 20;7:1709985. doi: 10.3389/fgwh.2026.1709985 (PMC13047111; doi:10.3389/fgwh.2026.1709985)
Supplement: SUPPLEMENTARY FILE 1 — Search strategies. [file Table1.docx]

**Searching strategies**

| **Pub-med** | | |  |
| --- | --- | --- | --- |
| **No** | **Search term** | **No** | **Date of searching** |
| 1 | ((((((((((((((((((((((((((((((((Climate change[MeSH Terms]) OR (Climate[Text Word])) OR ("global warming"[MeSH Terms])) OR (natural disaster[MeSH Terms])) OR ("Air pollution"[MeSH Terms])) OR (extreme weather[MeSH Terms])) OR (temperature[MeSH Terms])) OR (environmental exposure[MeSH Terms])) OR (heat stress[MeSH Terms])) OR (droughts[MeSH Terms])) OR (wildfires[MeSH Terms])) OR (floods[MeSH Terms])) OR ("extreme temperature"[Text Word])) OR (precipitation[Text Word])) OR (wind[Text Word])) ) OR ("rainfall shocks"[Text Word])) ) OR ("Sea Level Rise"[Text Word])) OR (hurricanes[Text Word])) OR ("Climate crisis"[Text Word])) OR ("Climate disruption"[Text Word])) OR ("Environmental change"[Text Word])) OR ("Environmental degradation"[Text Word])) OR ("Environmental factors"[Text Word])) OR ("Climate anomaly"[Text Word])) OR (cyclones[Text Word])) OR ("Greenhouse Effect"[Text Word])) OR ("Cyclonic Storms"[Text Word])) OR (migration[Text Word])) OR ("resource scarcity"[Text Word]))) AND ((((((((((((((((((((((((abortion, spontaneous[MeSH Terms]) OR (abortion, induced[MeSH Terms])) OR (reproductive health[MeSH Terms])) OR (Abortion[Text Word])) OR (miscarriage[Text Word])) OR ("pregnancy loss"[Text Word])) OR ("spontaneous abortion"[Text Word])) OR ("fetal loss"[Text Word])) OR ("Habitual abortion"[Text Word])) OR ("pregnancy outcome"[Text Word])) OR ("induced abortion"[Text Word])) OR ("elective abortion"[Text Word])) OR ("abortion access"[Text Word])) OR ("abortion rates"[Text Word])) OR ("pregnancy termination"[Text Word])) OR ("legal abortion"[Text Word])) OR ("abortion care"[Text Word])) OR ("unsafe abortion"[Text Word])) OR ("access to abortion"[Text Word])) OR ("reproductive rights"[Text Word])) OR ("reproductive health"[Text Word])) OR ("abortion service"[Text Word])) | 6,600 | July 23, 2025. |
| **Embase(Ovid)** | | | |
| 2 | ((((((((((((((((((((((((((((((((exp "Climate change"/) OR (Climate.mp.)) OR (exp "global warming"/)) OR (exp "natural disaster"/)) OR (exp "Air pollution"/)) OR (exp "extreme weather"/)) OR (exp temperature/)) OR (exp "environmental exposure"/)) OR (exp "heat stress"/)) OR (exp droughts/)) OR (exp wildfires/)) OR (exp floods/)) OR ("extreme temperature".mp.)) OR (precipitation.mp.)) OR (wind.mp.))) OR ("rainfall shocks".mp.))) OR ("Sea Level Rise".mp.)) OR (hurricanes.mp.)) OR ("Climate crisis".mp.)) OR ("Climate disruption".mp.)) OR ("Environmental change".mp.)) OR ("Environmental degradation".mp.)) OR ("Environmental factors".mp.)) OR ("Climate anomaly".mp.)) OR (cyclones.mp.)) OR ("Greenhouse Effect".mp.)) OR ("Cyclonic Storms".mp.)) OR (migration.mp.)) OR ("resource scarcity".mp.))) AND ((((((((((((((((((((((((exp "abortion, spontaneous"/) OR (exp "abortion, induced"/)) OR (exp "reproductive health"/)) OR (Abortion.mp.)) OR (miscarriage.mp.)) OR ("pregnancy loss".mp.)) OR ("spontaneous abortion".mp.)) OR ("fetal loss".mp.)) OR ("Habitual abortion".mp.)) OR ("pregnancy outcome".mp.)) OR ("induced abortion".mp.)) OR ("elective abortion".mp.)) OR ("abortion access".mp.)) OR ("abortion rates".mp.)) OR ("pregnancy termination".mp.)) OR ("legal abortion".mp.)) OR ("abortion care".mp.)) OR ("unsafe abortion".mp.)) OR ("access to abortion".mp.)) OR ("reproductive rights".mp.)) OR ("reproductive health".mp.)) OR ("abortion service".mp.)))) | 6,198 | July 23, 2025. |
| **Ovid MEDLINE** | | | |
| 3 | ((((((((((((((((((((((((((((((((exp "Climate change"/) OR (Climate.mp.)) OR (exp "global warming"/)) OR (exp "natural disaster"/)) OR (exp "Air pollution"/)) OR (exp "extreme weather"/)) OR (exp temperature/)) OR (exp "environmental exposure"/)) OR (exp "heat stress"/)) OR (exp droughts/)) OR (exp wildfires/)) OR (exp floods/)) OR ("extreme temperature".mp.)) OR (precipitation.mp.)) OR (wind.mp.))) OR ("rainfall shocks".mp.))) OR ("Sea Level Rise".mp.)) OR (hurricanes.mp.)) OR ("Climate crisis".mp.)) OR ("Climate disruption".mp.)) OR ("Environmental change".mp.)) OR ("Environmental degradation".mp.)) OR ("Environmental factors".mp.)) OR ("Climate anomaly".mp.)) OR (cyclones.mp.)) OR ("Greenhouse Effect".mp.)) OR ("Cyclonic Storms".mp.)) OR (migration.mp.)) OR ("resource scarcity".mp.))) AND ((((((((((((((((((((((((exp "abortion, spontaneous"/) OR (exp "abortion, induced"/)) OR (exp "reproductive health"/)) OR (Abortion.mp.)) OR (miscarriage.mp.)) OR ("pregnancy loss".mp.)) OR ("spontaneous abortion".mp.)) OR ("fetal loss".mp.)) OR ("Habitual abortion".mp.)) OR ("pregnancy outcome".mp.)) OR ("induced abortion".mp.)) OR ("elective abortion".mp.)) OR ("abortion access".mp.)) OR ("abortion rates".mp.)) OR ("pregnancy termination".mp.)) OR ("legal abortion".mp.)) OR ("abortion care".mp.)) OR ("unsafe abortion".mp.)) OR ("access to abortion".mp.)) OR ("reproductive rights".mp.)) OR ("reproductive health".mp.)) OR ("abortion service".mp.)))) | 3,559 | July 23, 2025. |
| **PsycINFO(Ovid)** | | | |
| 4 | ((((((((((((((((((((((((((((((((exp "Climate change"/) OR (Climate.mp.)) OR (exp "global warming"/)) OR (exp "natural disaster"/)) OR (exp "Air pollution"/)) OR (exp "extreme weather"/)) OR (exp temperature/)) OR (exp "environmental exposure"/)) OR (exp "heat stress"/)) OR (exp droughts/)) OR (exp wildfires/)) OR (exp floods/)) OR ("extreme temperature".mp.)) OR (precipitation.mp.)) OR (wind.mp.))) OR ("rainfall shocks".mp.))) OR ("Sea Level Rise".mp.)) OR (hurricanes.mp.)) OR ("Climate crisis".mp.)) OR ("Climate disruption".mp.)) OR ("Environmental change".mp.)) OR ("Environmental degradation".mp.)) OR ("Environmental factors".mp.)) OR ("Climate anomaly".mp.)) OR (cyclones.mp.)) OR ("Greenhouse Effect".mp.)) OR ("Cyclonic Storms".mp.)) OR (migration.mp.)) OR ("resource scarcity".mp.))) AND ((((((((((((((((((((((((exp "abortion, spontaneous"/) OR (exp "abortion, induced"/)) OR (exp "reproductive health"/)) OR (Abortion.mp.)) OR (miscarriage.mp.)) OR ("pregnancy loss".mp.)) OR ("spontaneous abortion".mp.)) OR ("fetal loss".mp.)) OR ("Habitual abortion".mp.)) OR ("pregnancy outcome".mp.)) OR ("induced abortion".mp.)) OR ("elective abortion".mp.)) OR ("abortion access".mp.)) OR ("abortion rates".mp.)) OR ("pregnancy termination".mp.)) OR ("legal abortion".mp.)) OR ("abortion care".mp.)) OR ("unsafe abortion".mp.)) OR ("access to abortion".mp.)) OR ("reproductive rights".mp.)) OR ("reproductive health".mp.)) OR ("abortion service".mp.)))) | 495 | July 24, 2025. |
| **Web of Science** | | | |
| 5 | TS=(climate* OR "climate change" OR "global warming" OR "natural disaster" OR "air pollution" OR "extreme weather" OR "environmental exposure" OR "heat stress" OR droughts OR wildfires OR floods OR "extreme temperature" OR precipitation OR wind OR "rainfall shocks" OR "sea level rise" OR hurricanes OR "climate crisis" OR "climate disruption" OR "environmental change" OR "environmental degradation" OR "environmental factors" OR "climate anomaly" OR cyclones OR "greenhouse effect" OR "cyclonic storms" OR "resource scarcity")ANDTS=(abortion OR miscarriage OR "pregnancy loss" OR "spontaneous abortion" OR "induced abortion" OR "elective abortion" OR "abortion access" OR "abortion care" OR "abortion rate*" OR "pregnancy termination" OR "safe abortion" OR "unsafe abortion" OR "fetal loss" OR "habitual abortion" OR "reproductive health" OR "reproductive right*" OR "abortion service*") | 2,209 | July 24, 2025. |
| **Google Scholar** | | | |
| 6 | with all of the words: Climate Abortion Pregnancy  with the exact phrase: climate change OR global warming OR natural disaster OR air pollution OR extreme weather OR temperature OR environmental exposure OR heat stress OR droughts OR wildfires OR floods OR extreme temperature OR precipitation OR wind OR rainfall shocks OR sea level rise OR hurricanes OR climate crisis OR climate disruption OR environmental change OR environmental degradation OR environmental factors OR climate anomaly OR cyclones OR greenhouse effect OR cyclonic storms OR migration OR resource scarcity  with at least one of the words: abortion OR miscarriage OR pregnancy loss OR spontaneous abortion OR fetal loss OR habitual abortion OR pregnancy outcome OR induced abortion OR elective abortion OR abortion access OR abortion rates OR pregnancy termination OR legal abortion OR abortion care OR unsafe abortion OR access to abortion OR reproductive rights OR abortion service OR reproductive health | 106 | July 24, 2025. |
