# Supplementary material for: Impact of climate change on spontaneous abortion: a systematic review and meta-analysis
Source: Front Glob Womens Health. 2026 Mar 20;7:1709985. doi: 10.3389/fgwh.2026.1709985 (PMC13047111; doi:10.3389/fgwh.2026.1709985)
Supplement: SUPPLEMENTARY FILE 2 — Reasons for exclusion of studies. [file Table2.docx]

**Reason for Exclusion**

| **No** | **Author** | **Title** | **Reason for exclusion** |
| --- | --- | --- | --- |
| 1 | M. Fukuda, et al. | Climate change is associated with male: female ratios of fetal deaths and newborn infants in Japan | Irrelevant outcome or Exposure |
| 2 | Logie, C. H. et al. | Experiences of drought, heavy rains, and flooding and linkages with refugee youth sexual and reproductive health in a humanitarian setting in Uganda: qualitative insights | Irrelevant outcome or Exposure |
| 3 | RB Smith, et al. | Impacts of air pollution and noise on risk of preterm birth and stillbirth in London | Irrelevant outcome or Exposure |
| 4 | Y Cui, et al. | Associations of residential greenness exposure during early pregnancy with the risk of spontaneous abortion: the China Birth Cohort Study | Irrelevant outcome or Exposure |
| 5 | N Mohammadi Dashtaki, et al. | A case-crossover study of air pollution exposure during pregnancy and the risk of stillbirth in Tehran, Iran | Irrelevant outcome or Exposure |
| 6 | RJ Veenema et al. | Climate change-related environmental exposures and perinatal and maternal health outcomes in the US | Irrelevant outcome or Exposure |
| 7 | S Rekha et al*.* | Hot ambient temperature and its impacts on Adverse Pregnancy Outcome (APO)-A preliminary finding of women at outdoor workplaces | Irrelevant outcome or Exposure |
| 8 | Gilliland, Frank et al. | Air pollution exposure assessment for epidemiologic studies of pregnant women and children: lessons learned from the Centers for Children's Environmental Health and Disease Prevention Research | Irrelevant outcome or Exposure |
| 9 | A Haertel et al. | AMBIENT POOR AIR QUALITY FROM NEARBY WILDFIRES LINKED TO PREGNANCY LOSS AND INFANT RESPIRATORY PROBLEMS OF OUTDOOR LABORATORY- HOUSED MACAQUES | Irrelevant outcome or Exposure |
| 10 | AP Shah et al. | Associations of climatic factors with pregnancy loss in Nicosia, Cyprus | Unable to get full text |
| 11 | M Dastoorpoor, et al. | Prenatal exposure to ambient air pollution and adverse pregnancy outcomes in Ahvaz, Iran: a generalized additive model | Unable to get full text |
| 12 | M Dastoorpoor, et al. | Acute effects of air pollution on spontaneous abortion, premature delivery, and stillbirth in Ahvaz, Iran: a time-series study | Unable to get full text |
| 13 | J He et al. | Association between short-term exposure to ambient fine particulate matter & components and the risk of spontaneous abortion | Unable to get full text |
| 14 | J Wang et al. | Relationship between concentration of inhaled pollutants, sulfur dioxide and nitrogen dioxide and spontaneous abortion | Unable to get full text |
| 15 | B Ritz et al. | Ambient air pollution and adverse birth outcomes: Methodologic issues in an emerging field | Not the primary study |
| 16 | Bonzini, M. et al. | Exposure to air pollutants during pregnancy and outcomes at birth: An epidemiological study in lombardy, italy, 2004-2008 | Irrelevant outcome or Exposure |
| 17 | Mainolfi, M. B. et al. | Low-level exposure to air pollution and risk of adverse birth outcomes in Hillsborough County, Florida | Irrelevant outcome or Exposure |
| 18 | Olsson, D. et al. | Air pollution exposure in early pregnancy and adverse pregnancy outcomes: A register-based cohort study | Irrelevant outcome or Exposure |
| 19 | Pereda, Paula Carvalho et al. | Climate change impacts on birth outcomes in Brazil | Irrelevant outcome or Exposure |
| 20 | Huang, C. et al. | Ambient air pollution and adverse birth outcomes: A natural experiment study | Irrelevant outcome or Exposure |
| 21 | Arroyo, Virginia et al. | Impact of air pollution and temperature on adverse birth outcomes: Madrid, 2001-2009 | Irrelevant outcome or Exposure |
| 22 | Arthurs, O. J. et al. | Ambient Air Pollution and Adverse Pregnancy Outcomes in Wuhan, China | Irrelevant outcome or Exposure |
| 23 | Capobussi, M. et al. | Air Pollution Impact on Pregnancy Outcomes in Como, Italy | Irrelevant outcome or Exposure |
| 24 | Grabich, S. C. et al. | Impact of Hurricane Exposure on Reproductive Health Outcomes, Florida, 2004 | Irrelevant outcome or Exposure |
| 25 | Grippo, Alexandra et al. | Air pollution exposure during pregnancy and spontaneous abortion and stillbirth | Irrelevant outcome or Exposure |
| 26 | Lamichhane, D. K. et al. | Air pollution exposure during pregnancy and ultrasound and birth measures of fetal growth: A prospective cohort study in Korea | Irrelevant outcome or Exposure |
| 27 | Abdo, M. et al. | Impact of wildfire smoke on adverse pregnancy outcomes in Colorado, 2007-2015 | Irrelevant outcome or Exposure |
| 28 | C Zhang et al. | Individual ambient ozone exposure during pregnancy and adverse birth outcomes: exploration of the potentially vulnerable windows | Irrelevant outcome or Exposure |
| **29** | B. Smith R et al. | Impacts of air pollution and noise on risk of preterm birth and stillbirth in London | Irrelevant outcome or Exposure |
| 30 | Wang, J. et al. | Exposure to heat wave during pregnancy and adverse birth outcomes: An exploration of susceptible windows | Irrelevant outcome or Exposure |
| 31 | C Zhang et al. | The Association between Ambient Particulate Matters Pollutant and Spontaneous Abortion of the First Trimester of Pregnancy in Tehran | Irrelevant outcome or Exposure |
| 32 | Zhang, X. et al. | Mapping the Research Landscape of Climate Change and its Impact on Pregnancy and Neonatal Outcomes: A Bibliometric Analysis | Irrelevant outcome or Exposure |
| 33 | Nadia Mohammadi Dashtaki et al. | A case-crossover study of air pollution exposure during pregnancy and the risk of stillbirth in Tehran, Iran | Irrelevant outcome or Exposure |
| 34 | Wang, T. et al. | Association of Ambient Air Pollution and Temperature Exposure with Placental Abruption: A Nested Case-Control Study Based on Live Birth Registrations | Irrelevant outcome or Exposure |
| 35 | Veras, M. M. et al. | Impact of air pollution and climate change on maternal, fetal and postnatal health | Irrelevant outcome or Exposure |
| 36 | Logie, C. H. et al. | Climate change, resource insecurities and sexual and reproductive health among young adolescents in Kenya: a multi-method qualitative inquiry | Irrelevant outcome or Exposure |
| 37 | Li-Maloney, C. et al. | Pregnancy and extreme heat events: A rapid review of evidence related to health outcomes, risk factors and interventions | Irrelevant outcome or Exposure |
| 38 | Huang, K. et al. | Associations of ambient air pollutants with pregnancy outcomes in women undergoing assisted reproductive technology and the mediating role of ovarian reserve: A longitudinal study in eastern China | Irrelevant outcome or Exposure |
| 39 | Huang, K. et al. | Interaction of Extreme Temperature Events and Fine Particulate Matter Components on Pregnancy Outcomes in Women Undergoing Assisted Reproductive Technology: A National Longitudinal Study | Irrelevant outcome or Exposure |
| 40 | Howells, M. et al. | Climate change, evolution, and reproductive health: The impact of water insecurity and heat stress on pregnancy and lactation | Irrelevant outcome or Exposure |
| 41 | Billingsley, S. et al. | Climate change and getting pregnant: a full accounting of conceptions in Armenia and Tajikistan | Irrelevant outcome or Exposure |
| 42 | Young, K. et al. | Wildfire Smoke Exposure During Pregnancy: Consensus-Building to Co-Create a Community-Engaged Study | Irrelevant outcome or Exposure |
| 43 | Ranciere, Fanny et al. | Associations between heat wave during pregnancy and term birth weight outcomes: The PARIS birth cohort | Irrelevant outcome or Exposure |
| 44 | Nyadanu, S. D. et al. | Short-term effects of wildfire-specific fine particulate matter and its carbonaceous components on perinatal outcomes: A multicentre cohort study in New South Wales, Australia | Irrelevant outcome or Exposure |
| 45 | Li, Y. et al. | The causal effect of exposure to air pollution on risk of adverse pregnancy outcomes: A two-sample Mendelian randomisation study | Irrelevant outcome or Exposure |
| 46 | Jiang, P. et al. | Wildfire particulate exposure and risks of preterm birth and low birth weight in the Southwestern United States | Irrelevant outcome or Exposure |
| 47 | Fottrell, A. K. et al. | Ambient Air Pollution, Housing Context, and Birth Outcomes Among Wisconsin Mothers | Irrelevant outcome or Exposure |
| 48 | Essers, E. et al. | Ambient air temperature exposure and foetal size and growth in three European birth cohorts | Irrelevant outcome or Exposure |
| 49 | Conway, Francesca et al. | Climate change, air pollution and maternal and newborn health: An overview of reviews of health outcomes | Not primary study |
| 50 | C Rousseau et al. | Climate change and sexual and reproductive health | Irrelevant outcome or Exposure |
| 51 | S Husaini et al. | Case report: another burden to bear: the impacts of climate change on access to sexual and reproductive health rights and services in Bangladesh | Not primary study |
| 52 | V Mohan et al. | Sexual and reproductive health and rights, climate, environment and co-existence in Madagascar | Irrelevant outcome or Exposure |
