# Supplementary material for: Impact of climate change on spontaneous abortion: a systematic review and meta-analysis
Source: Front Glob Womens Health. 2026 Mar 20;7:1709985. doi: 10.3389/fgwh.2026.1709985 (PMC13047111; doi:10.3389/fgwh.2026.1709985)
Supplement: SUPPLEMENTARY FILE 3 — Quality assessment of the included studies. [file Table3.docx]

**Quality assessment of the included studies**

| Study | Selection | comparability | Outcome/  exposure | Total score  (* as 1 point) | Study quality |
| --- | --- | --- | --- | --- | --- |
| Hou, H. Y et al , 2013 | **** | ** | * | 7 | High |
| Thimonier.A. et al, 2014 | **** | ** | *** | 9 | High |
| Asamoah, Benedict et ,al 2017 | **** | ** | ** | 8 | High |
| Ha, S. et al, 2018 | *** | ** | ** | 7 | High |
| Gaskins.A. J. et al, 2019 | *** | ** | * | 6 | Moderate |
| Xue, T. et al, 2019 | *** | ** | ** | 7 | High |
| Sun, Xiaoli et al, 2019 | *** | ** | ** | 7 | High |
| Kornfield, M.S. et Al, 2022 | *** | ** | * | 6 | Moderate |
| Zhou, W. Z. et al, 2022 | *** | ** | * | **6** | Moderate |
| Das, S. et al, 2023 | *** | ** | *** | 8 | High |
| Hajdu,T. et al, 2023 | **** | ** | ** | 8 | High |
| Xu, Q. et al, 2023 | **** | ** | ** | 8 | High |
| Zhao, S. et,.al 2023 | *** | ** | ** | 7 | High |
| He, C. et al, 2024 | **** | ** | ** | 8 | **High** |
| Li, J. et al, 2024 | **** | ** | ** | 8 | High |
| Moodley, Y. et al 2024 | **** | ** | ** | 8 | High |
| K. Wesselink et al, 2023 | **** | ** | ** | 8 | High |
| Tong, M. et al, 2023 | **** | ** | ** | 8 | High |
| Jukic, A. M. Z. et al, 2025 | *** | ** | *** | 8 | High |
| Wesselink, A. K. et al 2024 | *** | * | *** | 7 | High |
| Rekha, S. et al 2023 | *** | ** | * | 6 | Moderate |
| Bogan M et,.al 2021 | *** | ** | *** | 8 | High |
| Davenport et al, 2020 | *** | * | *** | 7 | High |
| Di Ciaula A, et al, 2015 | *** | ** | * | 6 | Moderate |
| Wang B, et al, 2020 | *** | * | *** | 7 | High |
| Moridi M, et al, 2014 | *** | * | *** | 7 | High |
| Bianchi-Demicheli F, et al, 2001 | *** | * | *** | 7 | High |
| Enkhmaa D , et al, 2014 | *** | ** | * | 6 | Moderate |
| Khodadadi N , et al 2022 | *** | ** | ** | 7 | High |
| Zhang L , et al, 2019 | *** | ** | ** | 7 | High |
| Zhang Y, et al, 2019 | *** | ** | ** | 7 | High |
| Xue T et al, 2021 | *** | ** | ** | 7 | High |
| Leiser CL et al, 2019 | *** | ** | ** | 7 | High |
| Liang Z, et al 2021 | **** | ** | ** | 8 | High |
| Wang H, et al 2021 | **** | ** | ** | 8 | High |
| Qu, et al 2021 | **** | ** | ** | 8 | High |
| Dastoorpoor et al, 2021 | *** | ** | ** | 7 | High |
