# Supplementary material for: Impact of climate change on spontaneous abortion: a systematic review and meta-analysis
Source: Front Glob Womens Health. 2026 Mar 20;7:1709985. doi: 10.3389/fgwh.2026.1709985 (PMC13047111; doi:10.3389/fgwh.2026.1709985)
Supplement: SUPPLEMENTARY FILE 4 — Sensitivity analysis for high ambient temperature and particulate matter exposure. [file Table4.docx]

**Sensitivity analysis for high ambient temperature**

| **Study omitted** | **Estimate** | **LCI** | **UCI** |
| --- | --- | --- | --- |
| Asamoah Benedict. et al, 2017 | 1.3842348 | 1.3842348 | 1.3842348 |
| Sun Xiaoli. et al, 2019 | 1.3842348 | 1.1900835 | 1.6100602 |
| M. Dastoorpoor. et al, 2021 | 1.3842348 | 1.1900835 | 1.6100602 |
| Bogan M. et al 2021 | 1.3842348 | 1.1900835 | 1.6100602 |
| Qu. et al, 2021 | 1.4159639 | 1.2070186 | 1.6610793 |
| Khodadadi N. et al, 2022 | 1.3842348 | 1.1900835 | 1.6100602 |
| Rekha S. et al, 2023 | 1.3842348 | 1.1900835 | 1.6100602 |
| Tong M. et al, 2023 | 1.3772879 | 1.1776018 | 1.6108347 |
| Das S. et al, 2023 | 1.3842348 | 1.1900835 | 1.6100602 |
| Zhao S. et al, 2023 | 1.3842348 | 1.1900835 | 1.6100602 |
| Wesselink A.K. et al, 2024 | 1.3842348 | 1.1900835 | 1.6100602 |
| Moodley Y. et al, 2024 | 1.3842348 | 1.1900835 | 1.6100602 |
| Combined | 1.3842348 | 1.1900835 | 1.6100602 |

**Sensitivity analysis for particulate matter exposure**

| **Study omitted** | **Estimate** | **LCI** | **UCI** |
| --- | --- | --- | --- |
| Moridi M. et al, 2014 | 1.0777839 | 1.0479288 | 1.1084898 |
| Thimonier A. et al, 2014 | 1.1168034 | 1.034258 | 1.1010336 |
|  | 1.1168034 | 1.044258 | 1.1010336 |
| Di Ciaula A. et al, 2015 | 1.0685019 | 1.0392705 | 1.0985556 |
| Ha S. et al, 2018 | 1.0777839 | 1.0479288 | 1.1084898 |
| Gaskins AJ. et al, 2019 | 1.0728426 | 1.0426724 | 1.1038859 |
| Zhang L. et al, 2019 | 1.0686314 | 1.0386252 | 1.0995044 |
| Zhang Y. et al, 2019 | 1.0777839 | 1.0479288 | 1.1084898 |
| Bogan M. et al, 2021 | 1.0777839 | 1.0479288 | 1.1084898 |
| Wang H. et al, 2021 | 1.0454941 | 1.0227851 | 1.0687075 |
| Li J. et al | 1.0717193 | 1.0418156 | 1.1024814 |
| Combined | 1.077784 | 1.0479288 | 1.1084897 |
